# A core set of venom proteins is released by entomopathogenic nematodes in the genus *Steinernema*

**DOI:** 10.1371/journal.ppat.1007626

**Published:** 2019-05-01

**Authors:** Dennis Z. Chang, Lorrayne Serra, Dihong Lu, Ali Mortazavi, Adler R. Dillman

**Affiliations:** 1 Department of Nematology, University of California, Riverside, California, United States of America; 2 Department of Developmental and Cell Biology, Center for Complex Biological Systems, University of California, Irvine, California, United States of America; University of Pennsylvania, UNITED STATES

## Abstract

Parasitic helminths release molecular effectors into their hosts and these effectors can directly damage host tissue and modulate host immunity. Excreted/secreted proteins (ESPs) are one category of parasite molecular effectors that are critical to their success within the host. However, most studies of nematode ESPs rely on *in vitro* stimulation or culture conditions to collect the ESPs, operating under the assumption that *in vitro* conditions mimic actual *in vivo* infection. This assumption is rarely if ever validated. Entomopathogenic nematodes (EPNs) are lethal parasites of insects that produce and release toxins into their insect hosts and are a powerful model parasite system. We compared transcriptional profiles of individual *Steinernema feltiae* nematodes at different time points of activation under *in vitro* and *in vivo* conditions and found that some but not all time points during *in vitro* parasite activation have similar transcriptional profiles with nematodes from *in vivo* infections. These findings highlight the importance of experimental validation of ESP collection conditions. Additionally, we found that a suite of genes in the neuropeptide pathway were downregulated as nematodes activated and infection progressed *in vivo*, suggesting that these genes are involved in host-seeking behavior and are less important during active infection. We then characterized the ESPs of activated *S*. *feltiae* infective juveniles (IJs) using mass spectrometry and identified 266 proteins that are released by these nematodes. In comparing these ESPs with those previously identified in activated *S*. *carpocapsae* IJs, we identified a core set of 52 proteins that are conserved and present in the ESPs of activated IJs of both species. These core venom proteins include both tissue-damaging and immune-modulating proteins, suggesting that the ESPs of these parasites include both a core set of effectors as well as a specialized set, more adapted to the particular hosts they infect.

## Author summary

In this study we found a core set of 52 venom proteins conserved between two insect-parasitic nematodes *Steinernema feltiae* and *Steinernema carpocapsae*, that are released when initially exposed to host tissue. Most of these proteins are conserved in mammalian-parasitic nematodes suggesting that this core set of proteins is important for parasitic nematodes in general. We show that the relevance of *in vitro* model systems to *in vivo* model systems needs to be optimized and experimentally measured. Using an *in vitro* model of parasitic nematode activation, we stimulated protein release from *S*. *feltiae* and evaluated its activity *in vivo*. This activation model was previously developed using *S*. *carpocapsae* and we conclude that this method is robust and can be generalized to other EPNs (entomopathogenic nematodes). We found notable characteristics of *S*. *feltiae* venom including time-dependent decreases in protein amount and toxicity after exposure to host tissue, which differs from what has been previously reported for other EPNs, illustrating diversity in parasitic strategies among EPNs. Additionally, naïve *S*. *feltiae* infective juveniles (IJs) not exposed to host tissue release considerable amounts of protein. These proteins however are not toxic and differ in composition from those of activated IJs.

## Introduction

Parasitic nematodes continue to be a major source of mortality and morbidity worldwide, infecting nearly 25% of the global population [1, 2]. The molecules that are released by these parasites, including the excreted/secreted proteins (ESPs), represent the major interface between hosts and parasites, and directly influence the survival and health of the parasites as well as the pathology they cause to the hosts [3, 4]. Despite an abundance of studies addressing mechanistic aspects of host immune response to nematode parasites, there is a distinct paucity of molecular information about most parasitic nematodes, where few secreted molecules have been studied in detail. Further, the role of the parasite ESP composition in determining host specificity is unknown. What is known relies largely on ESP studies where release of the ESPs is stimulated and collected *in vitro*. An underlying assumption is that the ESPs collected under these conditions are relevant and similar to the ESPs released in *in vivo* infections, though this assumption has not been experimentally validated for most if not all such studies [3]. Obtaining enough ESPs from nematodes that are actively involved in a host infection for subsequent analysis is difficult if not impossible. However, sequencing the transcriptomes of individual nematodes [5, 6], provides a way of comparing transcriptional profiles of parasites undergoing *in vitro* activation and *in vivo* infection.

Entomopathogenic nematodes (EPNs) are parasites of insects that rapidly kill their hosts. When EPNs deplete host nutrients the developing generation emerges from the cadaver as infective juveniles (IJs), an alternative third-stage larval form (L3) that is developmentally arrested, similar to the dauer juvenile stage in *C*. *elegans* [7]. The IJs are the only free-living stage of these nematodes, and they actively seek hosts to infect [8, 9]. Upon entering a new host, the IJs undergo the process of activation, or recovery from dauer, which entails resumption of growth and development, along with changes in morphology and gene expression that facilitate transition from a free-living form to an actively parasitic form [5, 10–12].

EPNs are being used as models for host-parasite interactions including ecology [13, 14], host-seeking behavior [9, 15], neurobiology [8], parasite activation [5, 16, 17], and the role of secreted products in parasitism [5, 18, 19]. There are more than 70 described species of EPNs in the genus *Steinernema*, and these vary in their host range and specificity [20, 21], making these nematodes a potential model for understanding the evolution of ESPs and their role in niche partitioning among parasites. For example, *S*. *carpocapsae* is a generalist parasite capable of infecting more than 250 different species of insects from at least 13 orders [22, 23], while other species such as *S*. *scapterisci* and *S*. *scarabaei* are specialist parasites infecting a much narrower range of species [24, 25]. A recent study of the *S*. *carpocapsae* secretome found that this generalist parasite releases more than 450 different proteins when initiating active parasitism. Many of these proteins were hypothesized to be involved in tissue damage and immunosuppression of the host [5]. *S*. *feltiae* is another generalist EPN parasite but with a more limited host range than *S*. *carpocapsae* and in a different clade within *Steinernema* [26, 27]. Several studies have shown that *S*. *feltiae* IJs use their cuticle to suppress and evade host immunity [28–30]. It has even been postulated that unlike *S*. *carpocapsae*, *S*. *feltiae* does not use secretion processes or secreted proteins to induce host immunosuppression [31].

Here we utilized RNA-seq from individual *S*. *feltiae* nematodes throughout a time course of *in vitro* and *in vivo* activation to compare the induction of ESPs under these different conditions. We reported the secretome of *S*. *feltiae* and tested its activity *in vivo*. We showed that activated *S*. *feltiae* IJs release a variety of proteins likely involved in tissue damage as well as immune modulation. By analyzing the *in vivo* time course of activation, we identified putative neuropeptide pathway genes likely to be involved in host-seeking behavior as the expression of these genes decreased as the nematodes is activated. Further, using comparative analysis we identified a core suite of 52 ESPs released by both *S*. *feltiae* and *S*. *carpocapsae* during active parasitism, indicating that despite differences in host range and specificity, some proteins may be broadly useful in parasitizing insect hosts. Most of these core proteins are conserved in nematode parasites of mammals, suggesting that they have an important and conserved role in parasitism.

## Results

### Steinernematids initiate active parasitism when exposed to host tissue

**Fig 1:**
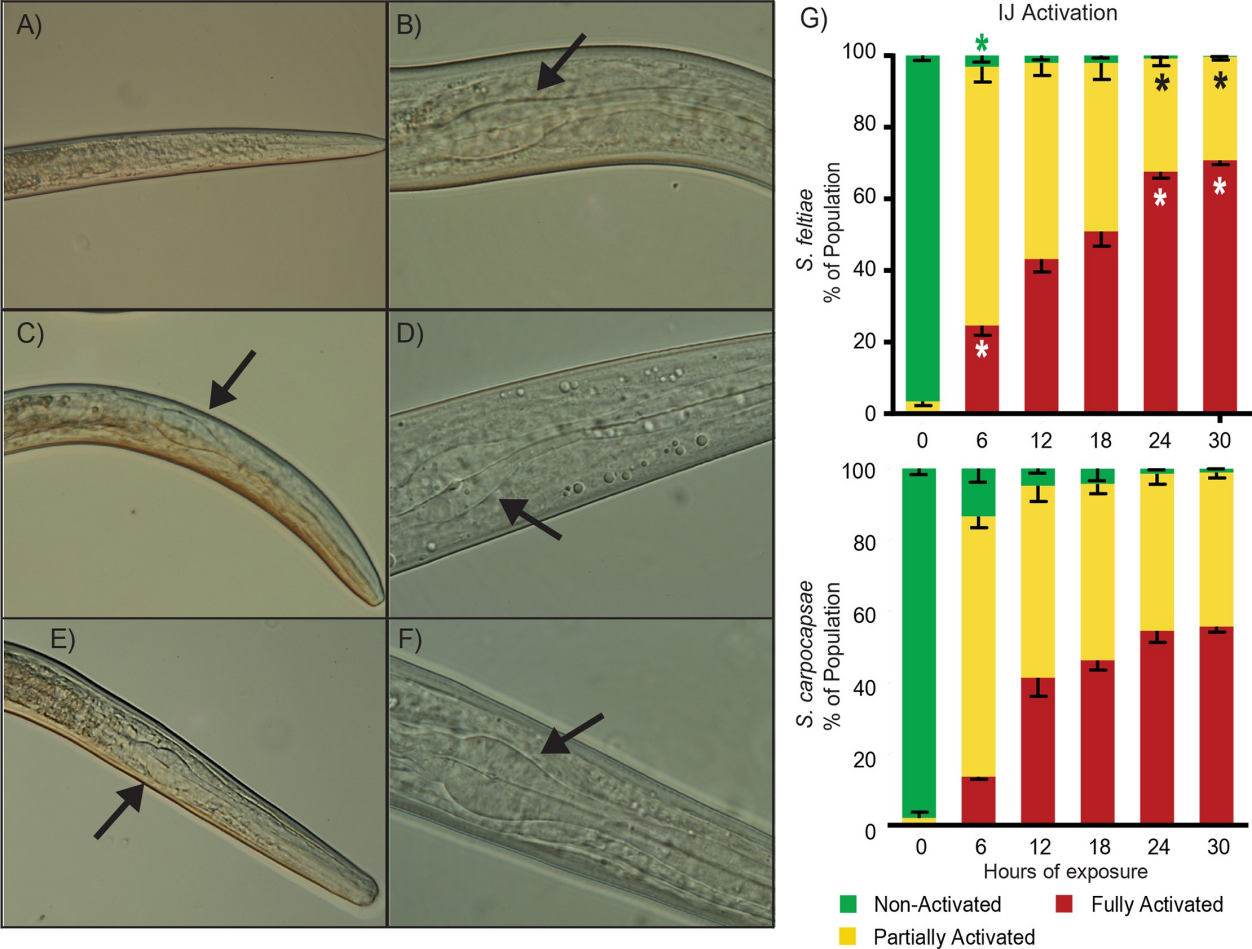
Activation of *S*. *feltiae* IJs. The left panel images are representative images of the head region of *S*. *feltiae* IJs exhibiting (A) naïve, (C) partially activated, and (E) fully activated morphology (400x). The pharyngeal bulb, if observable, is indicated by a black arrow. The right panel images are 1000x representative images of the *S*. *feltiae* IJs exhibiting activation morphology corresponding to the left panel images with (B) naïve, (D) partially activated, and (F) fully activated. (G) Time course activation rates based on activation morphology of IJs exposed to insect homogenate for 0, 6, 12, 18, 24, and 30 hours. All activation rate data was taken from IJs observed under 400x. The top graph is of *S*. *feltiae* activation and bottom graph is of *S*. *carpocapsae* activation (*S*. *carpocapsae* activation was reproduced from Lu. et al, 2017 with the addition of a 0-hour time point). Stars in the columns of the *S*. *feltiae* activation graph indicates a significant difference with p<0.05 between *S*. *feltiae* and *S*. *carpocapsae* rates of the same category (e.g. *S*. *feltiae* 6 hr full activation compared to *S*. *carpocapsae* 6 hr full activation, data in S1 Table). Column bars represent the mean with error bars representing standard deviation. Statistical analysis was done using a repeated measures two-way ANOVA with Sidak’s multiple comparisons test.

We utilized an *in vitro* activation method previously used for *S*. *carpocapsae* and *S*. *scapterisci* [5, 17] to determine how *S*. *feltiae* IJs activate. We exposed *S*. *feltiae* IJs to insect homogenate and found that they activated in a manner similar to what has been described for *S*. *carpocapsae* and *S*. *scapterisci* (Fig 1). Expansion of the pharyngeal bulb was found to be a reliable indicator of IJ activation [5, 16, 17] and this feature was used to quantify activation. In naïve IJs (IJs not exposed to host tissue) the pharyngeal bulb is often difficult to observe at 400x magnification (Fig 1A). At 1000x magnification (Fig 1B) the pharyngeal bulb can be seen, however the bulb is typically more compressed, seemingly deflated, when compared to activated nematodes. As IJs are exposed to host tissue over time they begin exhibiting partially-activated morphology characterized by partial expansion of the pharyngeal bulb (Fig 1D) which, in contrast to naïve IJs, is more expanded and can be readily observed at 400x (Fig 1C). These differences allow us to quickly and efficiently differentiate between non-activated and activated IJs under 400x magnification. After 6 hours of exposure to insect tissue, approximately 25% of IJs exhibit fully activated morphology with full expansion of the pharyngeal bulb, which is wider and appears rounder than the oval shape of partially activated nematodes (Fig 1F and 1D). Similar to what was observed for *S*. *carpocapsae*, *S*. *feltiae* exhibits high levels of activation (combined partial and full activation) after only 6 hours of exposure to host tissues (Fig 1G). However, *S*. *feltiae* IJs exhibited a higher percentage of fully activated morphology (approx. 25%) compared to *S*. *carpocapsae* (approx.15%) at 6 hours. And while both species displayed time-dependent increase in activation rates, *S*. *feltiae* activation rates were often higher than *S*. *carpocapsae* with significantly higher full activation rates after 6, 24, and 30 hours of exposure (Fig 1G, S1 Table).

### Activated *Steinernema* IJs release toxic proteins into their hosts

**Fig 2:**
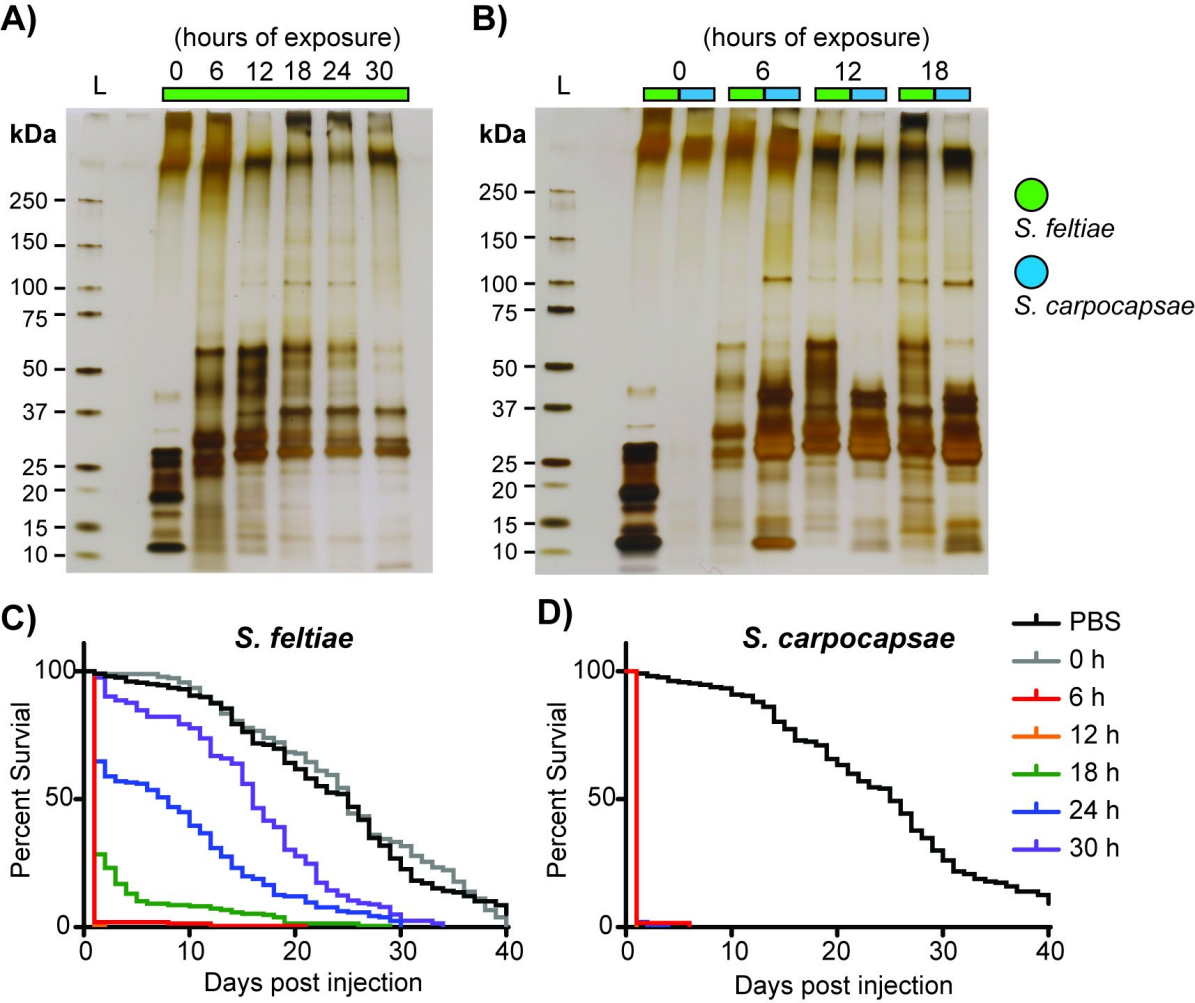
Steinernema IJs release toxic proteins. (A) Silver stained protein gel of whole ESPs collected from *S*. *feltiae* IJs activated for 0 (non-exposed), 6, 12, 18, 24, and 30 hours in insect homogenate. All time course activations were done with approximately 2.5 million IJs and the collected ESPs were concentrated to the same volume (300 μl) and the same volume (3 μl) was loaded to each lane. (B) Silver stained protein gel of whole ESPs (1 μg) from *S*. *feltiae* (green) and *S*. *carpocapsae* (blue) activated for 0, 6, 12, and 18 hours. (C) Survival curves of flies injected with 20 ng of whole ESPs from *S*. *feltiae*. (D) Survival curves of flies injected with 20 ng of whole ESPs from *S*. *carpocapsae* (*S*. *carpocapsae* survival curve was recapitulated from Lu. et al, 2017). Each survival curve includes 3 or more biological replicates totaling at least 180 flies.

After determining the activation dynamics of *S*. *feltiae* IJs, we collected the ESPs of activated *S*. *feltiae* IJs to determine their effect in insects. *S*. *feltiae* IJs were activated in insect homogenate for 0, 6, 12, 18, 24, or 30 hours, washed to remove the insect homogenate, and incubated in PBS for 3 hours where they continued releasing ESPs. The PBS (with accumulated ESPs) was then filtered through a 0.22 μm filter to remove the IJs and concentrated for further experiments. The relative age of all the ESPs were the same; at most, they were 3 hours old. We found that the profile of *S*. *feltiae* ESPs changed over time with proteins between 25 and 37 kDa being consistently present from 6–30 hours while proteins between 37–75 kDa peaked at 12 hours and diminished in abundance thereafter (Fig 2A). There was an overall time-dependent decrease in proteins released by *S*. *feltiae* (S1 Fig). Comparing the protein band profiles of *S*. *feltiae* and *S*. *carpocapsae* ESPs side-by-side shows that the majority of *S*. *feltiae* ESPs are between 25 and 75 kDa while *S*. *carpocapsae* ESPs are more concentrated in a narrower size range, between 25 and 50 kDa (Fig 2B). Naïve *S*. *feltiae* IJs produced a relatively large amount of ESPs, with most of these proteins below 37 kDa (Fig 2A and 2B) while naïve *S*. *carpocapsae* IJs produced undetectable levels of ESPs (Fig 2B).

Next, we tested the activity of *S*. *feltiae* ESPs in insect hosts. We injected 20 ng of *S*. *feltiae* ESPs into *Drosophila melanogaster* adults and monitored their survival. We found that the ESPs from naïve (0 hour) IJs were not toxic (Fig 2C). ESPs collected from the early activation time points (6 and 12 hours) exhibited the highest toxicity while ESPs from later activation time points (18, 24, and 30 hours) decreased in toxicity (Fig 2C). This activation-dependent toxicity is in stark contrast with *S*. *carpocapsae* ESPs, which maintained consistently high toxicity levels, even for ESPs collected after 30 hours of activation (Fig 2D). Late stage L4 and early adults were present at the later time points (24 and 30 hours) and since the more developed nematodes are fragile it was possible that some of these nematodes were damaged and unable to continue producing ESPs or were producing different ESPs. To address this possibility, we quantified the number of damaged nematodes throughout activation using a vital stain (0.2% trypan blue). Since it was the later time points (18, 24, and 30 hours) that exhibited notable decreases in ESP amount and toxicity we compared the number of damaged nematodes in these groups to that found among the 6-hour activated nematodes. The number of damaged nematodes did increase at the later time points (as expected) but the only group that exhibited a significantly higher percentage of damaged nematodes was the 30-hour time point, which accounted for less than 5% of the population (S2 Fig and S2 Table). Further, to simulate harsh experimental handling of the nematodes we repeated the activation time course but applied manual crushing/pressing of the activation sponge before washing the nematodes out for staining and observation. We found that manual crushing/pressing of the sponge caused significant increases in the percentages of damaged nematodes, with the highest average just below 12% at the 30-hour time point (S2 Fig and S2 Table). We also evaluated whether the toxicity we observed was primarily from nematode-derived ESPs or contamination from its symbiotic bacteria, *Xenorhabdus bovenii*. We compared ESPs from axenic *S*. *feltiae* IJs activated for 6 hours and found that the profile of ESPs and the toxicity (S3 Fig) were similar to those of symbiotic IJs (Fig 2A–2C), leading us to conclude that the toxicity in these experiments is a result of nematode-derived ESPs.

### Comparative transcriptome analysis of *in vitro* and *in vivo* activated *S*. *feltiae* IJs reveals a core set of genes expressed at 6 hours after activation

**Fig 3:**
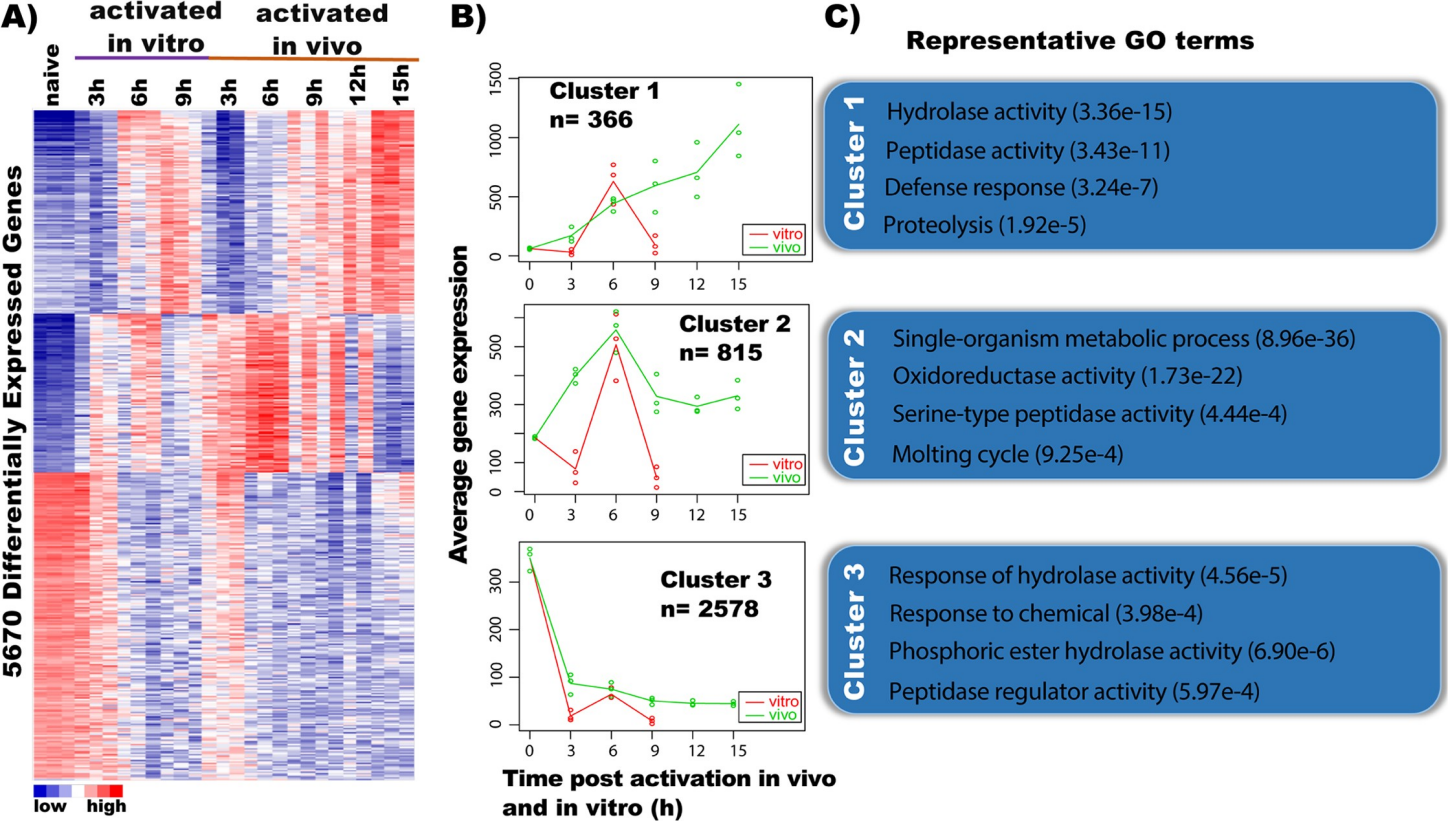
Genes differentially expressed during *in vitro* and *in vivo* IJ activation. (A) Heatmap showing the K-means of 5670 differentially expressed genes (FDR < 0.05) in activated IJs *in vitro* and *in vivo* using K = 3. (B) MaSigPro profiles of gene clusters during the time course (*in vitro* red, *in vivo* green). (C) Representative GO terms for each MaSigPro cluster.

We performed single-nematode RNA-seq analysis [6] in order to identify the similarities and differences between the activation of *S*. *feltiae in vivo* and *in vitro*. We collected RNA from 3 individual nematodes activated *in vitro* for 3, 6, and 9 hours and from nematodes dissected out of infected waxworms (*in vivo*) at 3, 6, 9,12, and 15 hours. We performed differential expression (DE) analysis using edgeR [32] and found 5670 genes to be differentially expressed between 6 hours *in vitro* activated IJs and naïve IJs (Fig 3A). Among these genes, 3 general gene expression patterns were observed: Increasing expression over time, increasing first and then decreasing over time, and high levels of expression in naïve IJs with expression decreasing over time (Fig 3A).

With the 5670 differentially expressed genes between 6-hour *in vitro* activated IJs and naïve IJs, we then used MaSigPro to identify genes with significant expression differences and similarities between *in vitro* and *in vivo* time courses and identified 3 major clusters (Fig 3B), similar to the result from edgeR analysis (Fig 3A) [33]. Cluster 1 consists of 366 genes that demonstrate a distinct profile between *in vitro* (red) and *in vivo* (green) conditions (Fig 3B). While the 6-hour *in vitro* and 6-hour *in vivo* samples had similar gene expression levels, many of these genes showed increasing expression up to 15 hours *in vivo*, whereas they showed decreasing expression by 9 hours *in vitro*. GO terms for defense response (p-value 3.24e-7), proteolysis (p-value 1.92e-5) as well as enzymatic activities such as peptidase (p-value 3.43e-11) and hydrolase (p-value 3.36e-15) are enriched in cluster 1 (Fig 3C). Enzymatic activity is also a feature of cluster 2 (815 genes) with enzymes such as oxidoreductase (p-value 1.73e-22) and serine-type peptidase (p-value 4.44e-4) reaching a peak of expression at 6 hours *in vitro* and *in vivo*. Lastly, cluster 3 consists of 2578 genes that decrease within 3 hours of activation. GO analysis of cluster 3 genes found enrichments in terms involved with response to hydrolase activity (p-value 4.56e-5), response to chemical (p-value 3.98e-4) and enzyme activity such as phosphoric ester hydrolase activity (p-value 6.90e-6) and peptidase regulator activity (p-value 5.97e-4) (Fig 3C).

An analysis of changes in gene expression over the time course (3, 6, 9,12, and 15 hours post infection) of *in vivo* activation also identified 3 major patterns of expression or clusters (S4 Fig). Cluster 1 has 286 genes and GO terms for defense response (p-value 1.44e-5) and enzymatic activity such as hydrolase (p-value 4.01e-9) and peptidase (p-value 6.49e-9) (S4 Fig). Cluster 3 consists of 1,153 genes and GO analysis found enrichments in terms involved in enzymatic regulation such as negative regulation of catalytic activity (p-value 4.71e-4), regulation of serine kinase activity (p-value 2.21e-4) and regulation of protein phosphorylation (p-value 2.18e-5). Cluster 2 has 1,353 genes which have a high expression in IJs and a sharp decrease in gene expression by 3 hours with a minor peak at 6 hours (S4 Fig). GO analysis reveals enzymatic activity is also a feature of cluster 2 with enzymes such as kinase (p-value 7.89e-5) and phosphoprotein phosphatase (p-value 5.8e-4). Interestingly, the GO analysis is also enriched for neuropeptide signaling pathway (p-value 4.18e-10) (S4 Fig, Cluster 2). We investigated further into the neuropeptide pathway genes and found that L889_g32029 (*Sf-flp-21*), which is orthologous to *C*. *elegans flp-21* and is a neuropeptide important for host-seeking behavior [34], decreases 8-fold in expression (S4 Fig). Similarly, L889_g7374 (*Sf-flp-11*), which is an ortholog of *C*. *elegans flp-11*, demonstrates strong expression at the IJ stage but has the sharpest decrease by 15 hours (S4 Fig). Other neuropeptides such as L889_g30047 (orthologous to *C*. *elegans flp-3)*, L889_g15885 (orthologous to *C*. *elegans flp-18)*, L889_g27993 (orthologous to *C*. *elegans flp-14*) and L889_g32992 (orthologous to *C*. *elegans flp-7*) are highly expressed at the IJ stage and progressively decrease by 15 hours post infection (S4 Fig).

Overall, both *in vivo* and *in vitro* time courses showed significant downregulation of a set of naïve IJ genes within 3 hours as well as equivalent activation of another set of genes by 6 hours and differentially express similar sets of genes associated with proteolytic enzymes (peptidases). The *in vivo*-only analysis is similar to the *in vivo* and *in vitro* DE analyses for both clusters 1 and 3 but have a different profile for cluster 2. In cluster 2 of the *in vivo*-only time course there is a decrease in the expression of neuropeptides (including ones thought to function in host-seeking behavior) at the later time points, which is likely correlated with reduction of host-seeking sensory functions after successful infection of a host.

### Protein components of *Steinernema* ESPs

Because of the high toxicity of the ESPs collected at the 6-hour time point and the similarity in gene expression between 6-hour *in vitro* and *in vivo* activated IJs, we chose to primarily focus on the 6-hour ESPs along with further analysis of ESPs from naïve IJs. Using mass spectrometry, we identified 266 proteins (False Discovery Rate, FDR < 5%, S3 Table). To determine the level of correlation between gene expression and relative protein abundance, an mRNA abundance (TPM, transcripts per million) to protein abundance (emPAI, exponentially modified protein abundance index) correlation analysis of the 266 proteins was performed. We found a weak positive correlation between mRNA and protein abundance with Pearson’s correlation value of 0.452 and Spearman’s rank value of 0.438 (S5 Fig).

**Fig 4:**
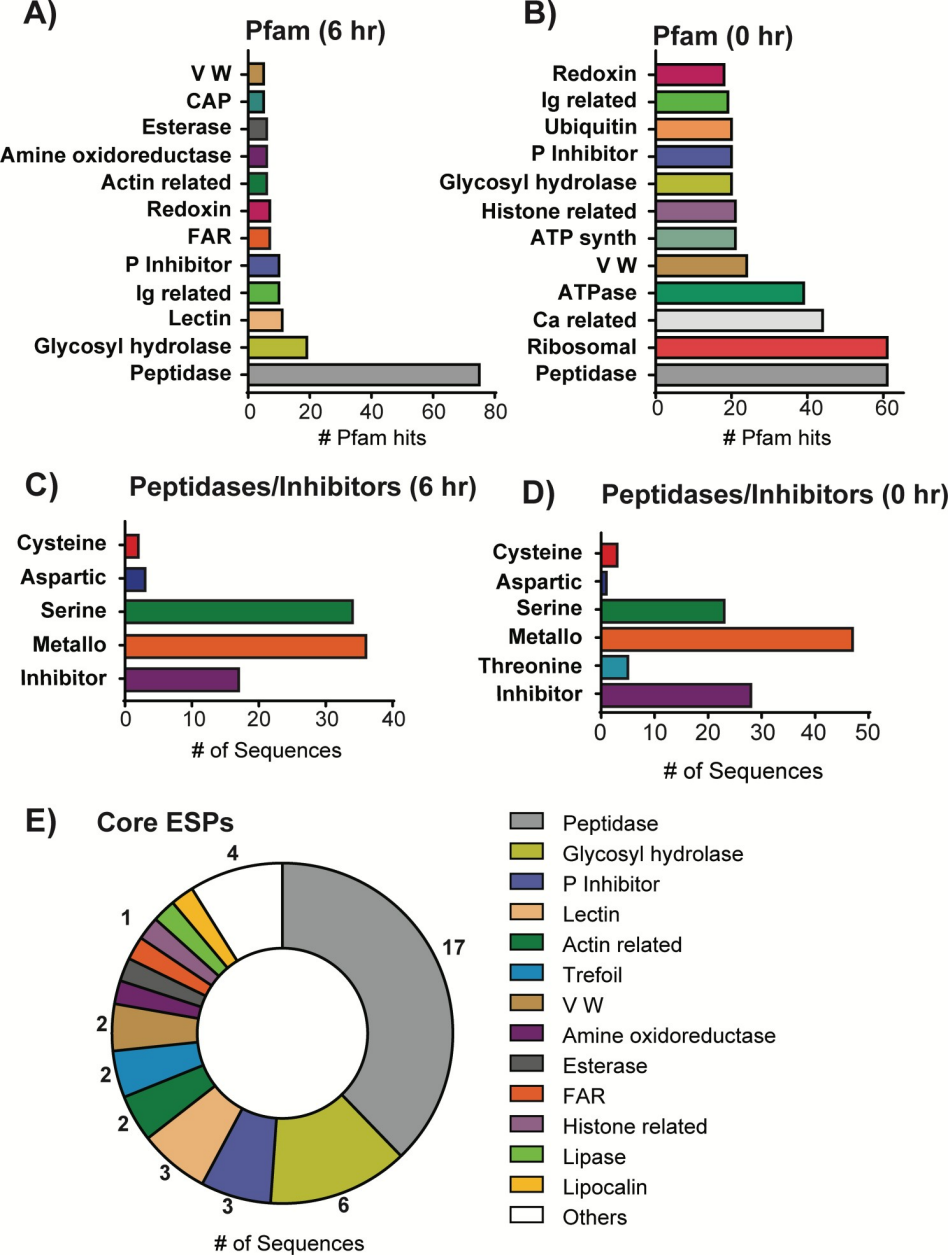
Protein components of *S*. *feltiae* and *S*. *carpocapsae*. Top 12 most abundant Pfam protein domains (E-value < 10^−5^) detected in ESPs of (A) *S*. *feltiae* activated for 6 hours and (B) *S*. *feltiae* naïve (0 hr) IJs. Peptidases and inhibitors detected using the MEROPS peptidase database for (C) *S*. *feltiae* activated for 6 hours and (D) *S*. *feltiae* naïve IJs (E-value < 10^−5^). (E) Pfam domains of core ESPs released by both *S*. *feltiae* and *S*. *carpocapsae* (E-value < 10^−5^).

We then analyzed the protein sequences for protein domains using Pfam, an online database of protein families [35]. Fig 4A lists the 12 most abundant Pfam domains in *S*. *feltiae* ESPs with peptidase domains being the highest in abundance followed by glycosyl hydrolases, lectins, Ig-related (Immunoglobulin like), and peptidase inhibitors. VW (Von Willebrand) domains and FAR domains were also found in relatively higher abundance (Fig 4A). A Merops (peptidase and peptidase inhibitor database) analysis detected 92 peptidases and 17 peptidase inhibitors with metallo and serine peptidase being the highest in abundance (Fig 4C). In analyzing the ESPs of naïve IJs we identified 682 proteins (FDR < 5%, S3 Table Sheet 2). Peptidase domains were also the highest in abundance in the ESPs from naïve IJs, followed closely by ribosomal, Ca-related (calcium interacting/regulating proteins) and ATPases (Fig 4B). A Merops analysis detected 79 peptidases and 28 inhibitors with both metallo and serine peptidases in high abundance; with the number of metallo peptidases more than double of serine peptidases (Fig 4D).

### Comparison of *S*. *feltiae* and *S*. *carpocapsae* secreted venom proteins reveals a small set of conserved catalytic enzymes

**Fig 5:**
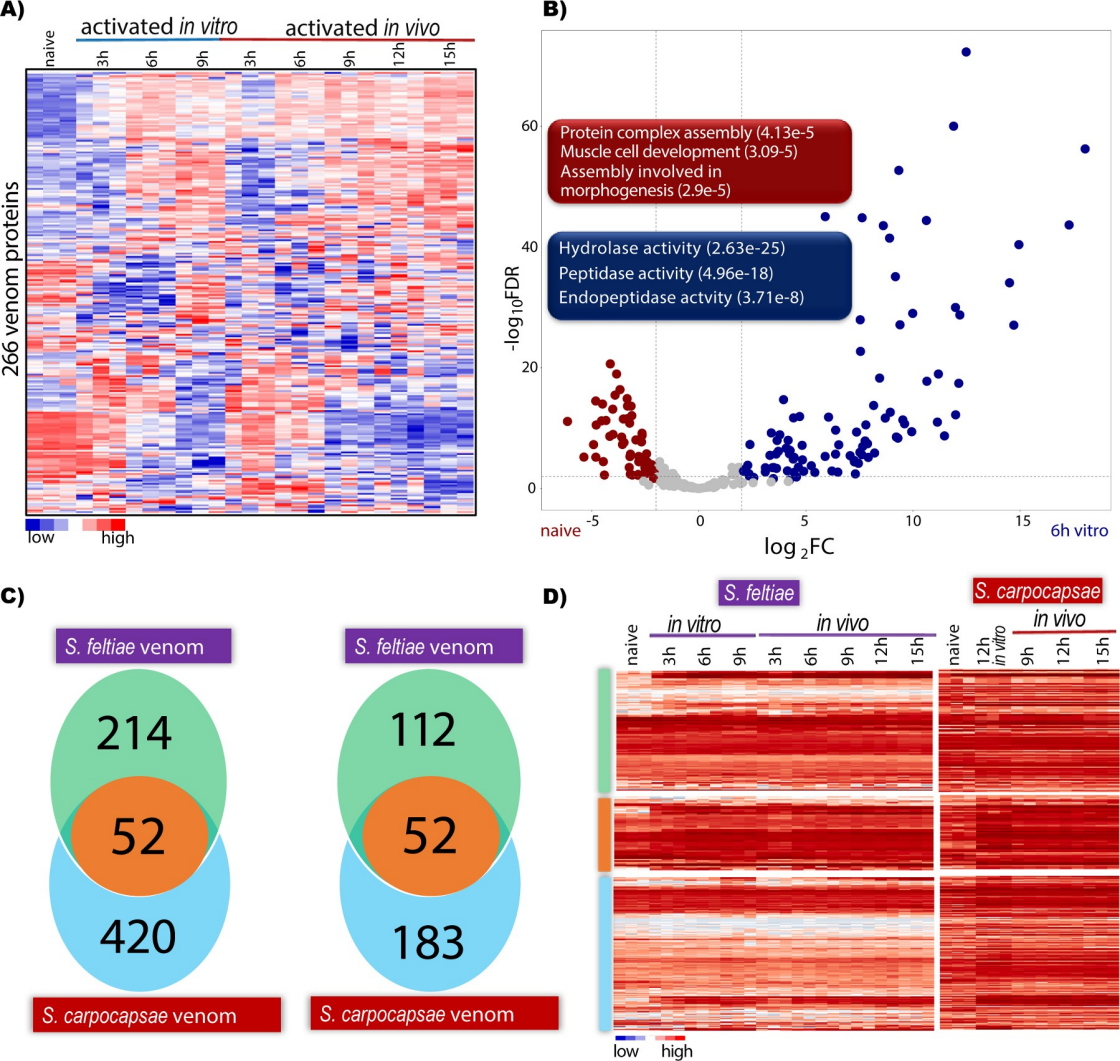
Gene expression of *S*. *feltiae* venom proteins *in vitro* and *in vivo* and comparison with *S*. *carpocapsae*. (A) Heatmap of the expression levels of 266 venom protein genes in both *in vitro* and *in vivo* activated IJs. (B) Volcano plot of 266 venom proteins showing the differentially expressed genes in non-activated and 6 hours *in vitro* activated IJs. Red and blue boxes are representative GO terms for significantly differentially expressed venom proteins. (C) Venn diagram illustrating the comparison of all *S*. *carpocapsae* venom proteins with *S*. *feltiae* venom proteins. (D) Venn diagram of conserved venom proteins with homologs in both species. 52 conserved proteins were detected in the venom of both species. (E) Heatmap of expression of the conserved homologs in panel D.

We confirmed that the mRNA of the 266 *S*. *feltiae* ESPs were detected at the 6-hour *in vitro* time point, and that these are expressed similarly at 6, 9, 12, and 15 hours *in vivo* (Fig 5A). We compared the gene expression of these 266 proteins between 6 hours *in vitro* and naïve IJs and found that 54 genes are downregulated and 96 genes are upregulated upon activation (Fig 5B). Gene ontology terms (GO) for the 96 upregulated genes show strong enrichment for enzymes such as hydrolases (p-value 2.63e-25) and peptidases (p-value 4.96e-18) and endopeptidase (p-value 3.71e-8), indicating that the activated nematodes increase the synthesis and release of enzymes to degrade host components, including proteins, at early stages of infection. In contrast, the 54 downregulated genes are related to muscle cell development (p-value 3.09e-5), protein complex assembly (p-value 4.13e-5) and morphogenesis (p-value 2.96e-5). These data suggest that at 6 hours *in vitro* the nematodes are at peak production of venom proteins.

We then conducted a comparative gene expression analysis of ESPs from *S*. *feltiae* and *S*. *carpocapsae* to understand the similarities and differences of genes involved in killing hosts. Our orthology analysis between 266 ESPs in *S*. *feltiae* and 472 *S*. *carpocapsae* found 52 genes in common (Fig 5C, S4 Table). This is a lower number than expected, given that 112 of the 266 *S*. *feltiae* ESPs have homologs in *S*. *carpocapsae* (S5 Table) and 183 of 472 ESPs found in *S*. *carpocapsae* have homologs in *S*. *feltiae* (Fig 5D, S6 Table). However, most of these homologs are not detected in the ESPs of the other species even when they are expressed (Fig 5E) suggesting that these enzymes might have been coopted over time to become part of the venom of either species. Interestingly, both *S*. *feltiae* and *S*. *carpocapsae* have a high expression of the shared 52 genes. GO terms analysis of the 52 genes shows enrichment of peptidases (p-value 1.25e-7), hydrolases (p-value 6.71e-10) and alpha-glucosidase activity (p-value 2.36e-5) (S7 Table). These results correlate with Pfam domains found in common between *S*. *feltiae* and *S*. *carpocapsae* (Fig 4A and [5]). We conclude that this small set of proteins form part of a core of venom proteins within *Steinernema*. Next, we wanted to determine whether these 52 ESPs from insect-parasitic nematodes were conserved in nematode parasites of vertebrates. We ran blastp on the 52 proteins (E-value < 1e-3) and compiled the best non-*Steinernema* hits for each protein. More than half (31 out of 52) of these genes have orthologs in mammalian-parasitic nematodes (S6 Fig) that include *Strongyloides ratti*, *Toxocara canis*, and *Ancylostoma duodenale* (S4 Table). The prevalence of these proteins in both insect- and vertebrate-parasitic nematode species leads us to speculate that these proteins may play critical roles during host infection and survival within the host for parasites in general.

## Discussion

### Activation of infective juveniles

Many nematodes have an alternate L3 stage of development, known as the dauer juvenile in free-living and necromenic species, or the infective juvenile for parasitic species [10, 36, 37]. The transition that parasitic IJs make when they enter a host and become actively parasitic and resume development is known as dauer recovery or activation. For parasitic nematodes, successful activation is critical to establishing a successful infection in their hosts [5, 11, 38, 39]. Similar to other EPNs, *S*. *feltiae* activation rates increased in a time dependent manner after exposure to insect tissue *in vitro* [5, 17]. After 30 hours of exposure to host tissue, essentially all the nematodes displayed some level of activation with non-activation rates being an average of 0.3% (S1 Table). Although *S*. *feltiae* and *S*. *carpocapsae* are in the same genus, they are members of different clades within the genus [27, 40, 41]. The fact that these EPNs display similar behavior and morphology during activation when exposed to insect tissue demonstrates that the *in vitro* model of activation we used is a consistent and robust model of activation. We found that when activated *in vitro*, the *S*. *feltiae* population does not exhibit synchronous activation. Some individuals are fully activated, some are partially activated, and a small number are not activated at all. We found this resolution of activation quantification to be reliable and consistent however we do note that these 3 categories of activation are broad; encompassing different degrees of pharyngeal bulb expansion, and that the resolution could have been increased by including other factors such as active pumping of the pharyngeal bulb or expansion of the anterior gut. Along with this phased activation, the full activation rates seem to taper off when the nematodes are activated for a long time (Fig 1G). Similar observations have been made for *S*. *carpocapsae* and *S*. *scapterisci* activation [5, 17]. The phenomenon of non-synchronous activation is similar to the phased infectivity reported in *in vivo* infections, wherein a certain percentage of an IJ population is unable to infect insect hosts or displays reduced infectivity, but over time more individuals become infectious [42, 43]. This characteristic is believed to be inherent to the IJ itself and does not seem to be significantly affected by factors such as IJ population or host population density. Studies have shown that phased infectivity correlates well with *Heterorhabditis* EPNs but not as well with *Steinernema* EPNs [44, 45]. In contrast to *H*. *bacteriophora*, where the infectious percentage of the population seems to start out low, previous research suggests that a large percentage of a *Steinernema* IJ population is typically infectious [44]. It has been suggested that the phased infectivity hypothesis is incomplete, and many other factors, such as genetic/physical damage, attraction to infected vs non-infected hosts, and survival of the IJ within the host, could affect population infectivity [46]. The age of the IJs could also be a contributing factor and was previously shown to affect activation rates in steinernematids [16]. In our *in vitro* model, the IJs do not actually infect a host, but rather are exposed to host tissue as if they had already infected the host. In this context, all the IJs are exposed to host tissue at the same time and though the majority of the population activate to some degree some individuals seem to respond faster and become fully activated early on while another portion of the population activates slower. We did not test whether population density was a factor, nor did we strictly control for age (IJs were between 2 weeks and 2 months post collection) but our findings are consistent with previous studies of phased infectivity. Thus *in vitro* activation may be a useful tool in further exploring the potential relationship between infectivity and activation.

### Using *in vitro* activation to study *in vivo* infection

It is widely recognized that helminths modulate host immune system and cause pathology mainly through the release of proteins and small molecules that interact with host cells and tissues, and that these molecules are key factors in disease pathology and parasite fitness [47, 48]. However, nearly all previous and current helminth secretome and ESP studies have been done *in vitro*, due to the difficulty of detecting ESPs from helminth parasites in their hosts. Additionally, there has been little if any experimental validation that the *in vitro* induction of ESPs from various parasitic helminths accurately mimics *in vivo* conditions.

Here, we utilized single-nematode RNA-seq to compare the transcriptomes of nematodes dissected out of waxworms after infection for 3, 6, 9, 12, and 15 hours and those of nematodes activated *in vitro* for 3, 6, and 9 hours. We found that the transcriptional profiles of nematodes activated *in vitro* were generally similar to those of nematodes from *in vivo* infections at each time point (Fig 3A) however some time points were more similar than others. We identified three major clusters of genes among the 5670 differentially expressed genes between activated and naïve IJs and within these three clusters the transcriptome profiles of the 6 h *in vitro* and 6 h *in vivo* activated nematodes exhibited the most consistent correlation (Fig 3B). In contrast, the gene expression profiles of nematodes activated *in vitro* and *in vivo* at 3h and 9h had significantly different profiles and did not correlate consistently (Fig 3B). Therefore, 3h and 9h *in vitro* are not representative of their *in vivo* counterparts. These data suggest that (1) activation of IJs *in vitro* can mimic *in vivo* infection and yield physiologically relevant results; (2) the fidelity of the *in vitro* results needs to be experimentally validated rather than simply assumed; and (3) selection of the timing of ESP collection should be based on the experimental evidence of when the *in vitro* system best mimics the natural process. It is important to determine the similarity of expression profiles for other parasites such as mammalian-parasitic nematodes freshly dissected from hosts compared to those stimulated under *in vitro* ESP collection conditions [49–51]. RNA-seq of individual nematodes, as we have done in this study, can be used to determine the similarity in the nematodes’ response to *in vitro* and *in vivo* conditions in order to optimize experimental *in vitro* conditions. This method is especially beneficial in parasitic studies where low parasite yield is a limiting factor. In addition, gene expression similarity should be optimized when using non-natural hosts, which are often used due to the difficulty of obtaining or maintaining natural hosts or lack of tools and techniques in non-model hosts compared to a model hosts such as a mouse.

### EPNs release lethal venom during infection

In EPN research, the nematode has been traditionally assumed to act primarily as a vector for the pathogenic bacterial symbiont. Once the bacterial pathogen is inside the host, it will kill the host while multiplying and providing nourishment (the bacteria itself and the insect tissue) for the nematode [10, 20, 52]. However, there is a growing body of research establishing the nematode as an active contributor to pathogenesis, and in some cases such as with *S*. *scapterisci*, the nematode may be the main driver of virulence [53]. It is clear that aside from serving as a vector for the bacteria they carry, EPNs contribute to pathogenesis in two ways: They directly damage host tissue and they dampen host immunity, acquiring more time for themselves and the bacteria they carry to overcome and kill the host. Past studies have shown that axenic *S*. *carpocapsae* IJs can kill and reproduce in insect hosts [54–56] and individual effector molecules from steinernematids have been characterized and shown to function in host immune suppression and tissue damage [18, 19, 57–61]. More recently the secretome of *S*. *carpocapsae* was shown to be a complex mixture containing many proteins and that collectively, this venom is toxic to insects. ESPs collected from axenic *S*. *carpocapsae* IJs had similar protein profiles as those from IJs associated with their bacterial symbiont, and the ESPs from both populations were similarly toxic [5]. We have shown these findings to also be true for *S*. *feltiae*, where *S*. *feltiae* IJs exposed to insect tissue become activated and produce ESPs (Fig 2A) that are toxic to insects (Fig 2C). ESPs collected from axenic *S*. *feltiae* IJs also displayed similar protein profiles and toxicity (Fig 2A and 2C; S3 Fig) compared to their symbiotic counterparts. For EPNs in the genus *Steinernema*, the nematodes seem to play a much more active role in contributing to pathogenicity during infection than previously thought.

We found that there are notable differences in ESP production and content among steinernematids. Whereas the protein profiles of *S*. *carpocapsae* ESPs were previously shown to be fairly constant after 6 to 30 hours of exposure to insect tissue [5] we found that the protein profiles and protein amount of *S*. *feltiae* ESPs change from 6 hours to 30 hours of exposure to host tissue (Fig 2A; S1 Fig). Comparing the profiles of ESPs from *S*. *feltiae* and *S*. *carpocapsae* side by side (Fig 2B), both have bands that are similar in size however the majority of intense *S*. *carpocapsae* bands are concentrated between 25–50 kDa while the majority of intense *S*. *feltiae* bands are not as concentrated and distinctly more spread out between 25–75 kDa. We found that there is a core suite of proteins found in the ESPs of both species and the differences in the protein profiles could be a result of adaptation to different bacterial symbionts or perhaps a result of host specialization. Another striking difference in ESP production between the two species is that when measuring ESPs from naïve IJs, *S*. *carpocapsae* was shown to produce few if any ESPs (not detectable by Bradford assay nor any notable bands by silver-staining (Fig 2B)) while naïve *S*. *feltiae* IJs produce a relatively large quantity of ESPs (Fig 2B). ESPs from naïve *S*. *feltiae* IJs shared some similarities with those from 6-hour activated IJs; namely that they were produced in relatively large quantities and included peptidases, peptidase inhibitors, and glycosyl hydrolases (Fig 4B). However, the protein profiles are different from each other (Fig 2A) where ESPs from naïve IJs contain a more diverse array of proteins (S3 Table sheet 2) and there were generally more peptides detected for each protein domain (Fig 4B). Further, the ESPs of naïve IJs were not toxic unlike their activated counterparts (Fig 2C).

The release of ESPs from naïve *S*. *feltiae* IJs without any stimulation from host cues seems metabolically wasteful. We evaluated the possibility that the ESPs from naïve IJs we collected were a result of damage from experimental handling rather than active release by the nematodes. We concluded that the contribution of ESPs from damaged nematodes is likely minimal for the following reasons: (1) *S*. *feltiae* IJs were treated exactly as *S*. *carpocapsae* IJs in a previous report [5], yet naïve *S*. *carpocapsae* IJs did not release detectable amounts of protein. (2) The nematodes in these experiments, if exposed to host tissue, began producing ESPs with a considerably different composition than naïve IJs (Figs 2B, 4A and 4B). (3) If allowed, the nematodes continued to develop into healthy, reproductive adults. Instead, our data reveals that naïve *S*. *feltiae* IJs are capable of producing a different set of ESPs, which could be involved with survival strategies including stress tolerance, lubrication and avoidance of desiccation, or maintaining the cuticle and other bodily structures. These strategies may be more pertinent to *S*. *feltiae* as it is categorized as more of a cruiser where it actively migrates in the soil seeking new hosts, while ambushers like *S*. *carpocapsae* tend to wait in epigeal habitats [13, 15]. Another possibility for the role of naïve *S*. *feltiae* IJ ESPs is preparation of the IJ cuticle for host infection since the cuticle of *S*. *feltiae* IJs has suppressive effects against host immune responses [28, 30, 31]. Peptidases, peptidase inhibitors, and immunoglobin-like proteins are detected in high abundance in the ESPs and they can be produced to potentially coat/adhere to the cuticle. The production of ESPs from naïve *S*. *feltiae* IJs is an interesting find that differentiates *S*. *feltiae* from *S*. *carpocapsae* and merits further study to understand the biology of this parasite.

The toxicity of activated *S*. *feltiae* ESPs was highest at the earliest time points tested (6 and 12 hours of exposure) and toxic activity decreased in a time-dependent manner with those collected after 24 and 30 hours of exposure being significantly less toxic (Fig 2C). The change in protein profiles (Fig 2A) and the reduced protein levels (S1 Fig) in *S*. *feltiae* ESPs over time seem to be correlated with the time-dependent toxicity decrease. However, it is unlikely that the reduction of toxicity is due to the decreasing abundance of total ESPs since the flies were exposed to the same amount of ESPs (20 ng per fly); instead, it is more likely that some low abundance toxin(s) in the mixture decrease(s) over time, resulting in lower toxic activity. The correlation between protein profiles/abundance and toxicity was not observed for *S*. *carpocapsae* ESPs: Later time points (42 and 54 hours of exposure) had similar protein profiles and protein abundance compared to earlier time points (6–30 hours of exposure), but were significantly reduced in toxicity or were not toxic at all [5]. This suggests that the toxic activity is due to low abundance proteins. Therefore, the toxins of both species are likely low abundance proteins and not the most abundant ones (Fig 2A and 2B). Other proteins found in the ESPs likely have non-toxic functions during infection such as immunosuppression or immune evasion.

We considered the possibility that damaged nematodes could be an explanation for the time-dependent decrease in ESP amount or toxicity and upon evaluation found a time correlated increase in the number of damaged nematodes. However, the highest level of damage we observed was less than 5% of the total population (S2 Fig). Even manually crushing the activation arena to simulate excessive force averaged less than 12% of the nematodes being damaged. We believe that the percentage of damaged nematodes from our experimental handling alone is insufficient to explain the dramatic changes we see in *S*. *feltiae* IJ ESP production and activity. It could also be argued that instead of (or in conjunction with) the nematodes being significantly damaged, they become unhealthy at the later time points due to various factors such as depletion of resources. We acknowledge this possibility however, it is unlikely the limiting factor as this was not observed in *S*. *carpocapsae* [5]. Instead, the time-dependent decrease in toxicity and amount of *S*. *feltiae* ESPs compared to the much slower decrease in toxicity and amount of *S*. *carpocapsae* ESPs suggests that these nematodes utilize different strategies in establishing themselves as parasites. *S*. *feltiae* may have a stronger reliance on its bacterial symbiont, *X*. *bovienii*, in order to overcome and kill the host. Soon after activation and release of bacterial symbionts, the IJs may switch their priority from killing the host to survival, feeding, and development. Axenic *S*. *feltiae* IJs have been shown to be capable of killing insect hosts, however the studies are limited compared to studies of *S*. *carpocapsae* and they generally report reduced efficiencies [62, 63]. We found no difference in activity between ESPs from axenic compared with symbiotic *S*. *feltiae* IJs, however we tested the activity of the ESPs alone and did not examine the larger context of an actual insect infection. It is possible that differences in ESP profiles between *S*. *carpocapsae* and *S*. *feltiae* are involved in niche partitioning and differences in host range and specificity.

### Core EPN venom proteins

We found 266 proteins in *S*. *feltiae* ESPs which is significantly fewer than the 472 proteins that were detected in *S*. *carpocapsae* ESPs [5]. However, this difference may be due to the more fragmented nature of the available *S*. *feltiae* genome, which has an N50 of 47.5kb compared to the 300kb N50 of the *S*. *carpocapsae* genome [40] that was used in the previous study (N50 is the length of the shortest contig that together with all the longer contigs cover 50% of the genome assembly). Although it is likely that the ESPs from EPNs are complex mixtures containing many different classes of molecules, we focused on analyzing the proteins. The most abundant group of proteins in activated *S*. *feltiae* venom are peptidases with a high proportion of serine and metallopeptidases (Fig 4A and 4C). This is similar to what was previously reported in *S*. *carpocapsae* ESPs [5]. However, *S*. *carpocapsae* ESPs contained fewer metallopeptidases and significantly more serine peptidases. The high abundance of peptidases and peptidase inhibitors in the ESPs of both species illustrate the importance of these enzymes for EPNs as well as other parasites. Many studies have implicated their potential use in vaccine development and treatment [64–67]. Peptidases and peptidase inhibitors have been shown to have multiple functions in parasite pathogenesis including suppressing/evading host immune systems, host tissue damage, and parasite development [68]. Serine peptidases in particular have been suggested to be used by many parasites including *Trichinella spiralis*, *Ascaris suum*, and *Brugia malayi*, among others [69–71]. Some specific characterizations of nematode serine peptidase functions include collagen degradation, suppression of melanization, inhibition of blood clotting, and parasite sperm activation [72–74].

We analyzed the protein domains in the ESPs to determine the potential molecular functions of the proteins. For *S*. *feltiae*, the second most abundant protein domain after peptidases were domains associated with hydrolysis of glycosydic bonds. These enzymes are hypothesized to have many potential functions, including cleavage of glycosolated proteins and breakdown of structural components that contain glycosidic bonds, with many similarities to peptidases. Some of the other protein domains detected in higher abundance in both *S*. *feltiae* and *S*. *carpocapsae* ESPs are Ig (immunoglobulin) or Ig-like, Von Willebrand, and FAR (fatty acid/retinol binding protein). The fact that both EPN species have high representation of these domains in their ESPs suggests their importance for EPN success. It is likely that some of these proteins are involved in immunomodulation. For example, it has been hypothesized that FAR proteins affect immune signaling [75], and while this has been experimentally demonstrated in plants [76–78], it has yet to be shown in an animal system. *S*. *feltiae* has been shown to modulate insect immunity using its cuticle but the use of specific excreted/secreted proteins in immune modulation by *S*. *feltiae* would be a novel finding [28, 31].

Additionally, we evaluated the correlation between mRNA abundance and protein abundance for these ESPs. The correlation was weak but positive with a Pearson’s correlation of 0.452 and Spearman’s rank correlation of 0.438 (S5 Fig). mRNA-protein abundance correlations have consistently been weak in various studies including those involving nematodes [79, 80] and our data support this trend. The discrepancies between mRNA and protein abundance is likely due to post-transcriptional regulating systems that can include small non-coding RNAs and microRNAs which has been postulated before [79]. It has been pointed out that most studies of mRNA-protein abundance correlation have been focused on transcriptome-wide data and a study specifically focused on upregulated transcripts resulted in a higher distribution of strong correlations, but we did not evaluate this in the present study [81].

In examining the 266 ESPs released by *S*. *feltiae* and the 472 ESPs released by *S*. *carpocapsae*, we found 52 proteins conserved in the ESPs of both species. This was unexpectedly low since 112 of the 266 *S*. *feltiae* ESPs have homologs in *S*. *carpocapsae* and 184 of the 472 *S*. *carpocapsae* ESPs have homologs in *S*. *feltiae* (Fig 5D). Both *S*. *feltiae* and *S*. *carpocapsae* have a high expression of the shared 52 venom genes, representing a core of effector proteins shared by these EPNs. Within this core set of ESPs there are peptidases, glycosyl hydrolases, lectins as well as proteins likely to be involved in immune modulation such as FAR proteins, immunoglobulins, and immunoglobulin-like proteins. The specific functions of these core venom proteins are yet unknown, but their conservation between *S*. *carpocapsae* and *S*. *feltiae*, which are in different clades within the genus, suggests that they are important effectors of parasitism and function in a variety of insect hosts. The genus *Steinernema* is the oldest known lineage of EPNs, potentially coevolving with their insect hosts for ~350 million years [26]. Determining the functions of the proteins in this core suite of ESPs may elucidate important steps in the evolution of EPNs and even more broadly parasitic nematodes in general.

## Materials/methods

### Insects

*Galleria mellonella* (waxworms) were purchased from CritterGrub (www.crittergrub.com). Oregon-R *Drosophila melanogaster* flies were reared in round bottom 8 oz bottles with food medium (129.4 g/L dextrose, 7.4 g/L agar, 61.2 g/L corn meal, 32.4 g/L yeast, and 2.7 g/L tegosept). The bottles were kept at 25°C with 60% relative humidity on a 12 hr light/dark cycle.

### Nematodes

*S*. *feltiae* IJs were cultured and propagated *in vivo* using waxworms as previously described [5]. Briefly,15 wax worms were placed into a 10 cm petri dish with filter paper pressed to the bottom and 1 ml of tap water containing 750 S. feltiae IJs (50 IJs/worm) was dispersed onto the filter paper. The infection plates were incubated at 25°C with 60% humidity in the dark for 10 days. Then, the waxworm cadavers were transferred to White traps [82]. After 2–3 days (depending on IJ density) the IJs were collected and washed using a glass vacuum filter holder (Fisher Scientific, 09-753-1C) with an 11 μm nylon mesh filter (Millipore, NY1104700). The IJs were stored at 15°C at a density of 7–10 IJs/μl.

### Waxworm homogenate preparation

Insect homogenate was prepared as previously described [5]. Briefly, 25 g of waxworms were frozen and grounded in liquid nitrogen with a mortar and pestle into a fine powder. The waxworm powder was then transferred quickly into a glass beaker and resuspended in 100 mL of Phosphate Buffered Saline (PBS, 137 mM NaCl, 2.7 mM KCl, 10 mM Na_2_HPO_4_, 1.8 mM KH_2_PO_4_, pH 7.4). The mixture was then microwaved to a boil 7–8 times with stirring in between. The homogenate was then aliquoted into 50 mL conical tubes and centrifuged for 5 minutes at 3200 rcf to pellet the solid debris of the waxworm. The supernatant, including the top oil layer were transferred into a new container. PBS was then added to the 50 mL conical tubes containing the waxworm pellets, mixed, centrifuged, and the supernatant was collected. This was repeated until the desired volume and percent extract was reached. In this case, 25g of waxworm was used to make 100 ml of 25% waxworm homogenate. The waxworm homogenate extract was used immediately or aliquoted and stored at -20°C.

### Activation of IJs

IJ activation was done as previously described [5]. 100 mL of 25% waxworm homogenate was thawed and supplemented with 1x triple antibiotic Pen/Strep/Neo (P4083, Sigma-Aldrich). The homogenate was soaked into 8.2 g of autoclaved cut sponge pieces (approximately 3x3x10 mm). 2.5 million *S*. *feltiae* IJs were washed 4 times with autoclaved 0.8% NaCl solution and excess liquid was removed from the washed IJs before gentle Pasteur pipette transferring/mixing into the homogenate-soaked sponge. The container was covered with aluminum foil and incubated in the dark at 25°C with 60% relative humidity for a specified amount of time. For most of the contents of this study, the IJs were incubated in waxworm homogenate for 6 hours. The IJs were then washed out of the sponge with 6–8 rounds of autoclaved 0.8% NaCl solution and once separated from the sponge, further washed with 4–5 rounds of 0.8% NaCl solution. Activations were replicated at least 3 times for each experiment.

### Quantification of activation rates

IJ activation quantification was done as described [5, 16, 17]. Briefly, activated IJs were observed under 400x magnification on a compound light microscope and scored for the activation phenotype based on expansions of the pharyngeal bulb. Fully activated phenotypes (see Fig 1E), partially activated phenotypes (Fig 1 image C), and Non-activated phenotypes (see Fig 1A) were scored. The difference between non-activated IJs from those that have been partially or fully activated is easily visualized as the absence of a visible pharyngeal bulb at 400x magnification. Differentiating between partially and fully activated IJs relies on the relative size and shape of the pharyngeal bulb; fully activated IJs have a wider, round-shaped bulb whereas partially activated IJs have a narrower, oval-shaped bulb. To minimize bias and double scoring the same nematode, scoring started with viewing IJs at one corner of the coverslip. All IJs with anterior/head region in view were scored before shifting the slide to view the next adjacent region. This was repeated until all regions of the coverslip was viewed without viewing the same region twice. Activations were done in 3 replicates for each time point (naïve/0 hr, 6 hr, 12 hr,18 hr, 24 hr, and 30 hr) and each replicate was scored 3 times to obtain averages. Significant differences between *S*. *feltiae* and *S*. *carpocapsae* IJ activations were determined using the Prism 8 by paired two-way ANOVA with (Prism recommended) Sidak’s multiple comparisons between related groups (i.e. rates of partially activated *S*. *feltiae* IJs at 30 hrs of exposure compared to rates of partially activated *S*. *carpocapsae* IJs at 30 hrs of exposure).

### ESP collection

ESP collection from the EPN was done as previously described [5]. After IJs were activated and thoroughly washed, they were transferred into a 1 L Erlenmeyer flask containing 100 mL of autoclaved PBS supplemented with 1x triple antibiotic Pen/Strep/Neo. The flask was shaken at 220 rpm in the dark for 3 hours and the nematodes were then centrifuged (700–800 rcf for 1 minute) in 15 mL conical tubes to preliminarily separate the majority of the nematodes from the PBS. The PBS supernatant was then collected and filtered through a 0.22 um syringe filter (Fisher Scientific, 9719001) and concentrated to approximately 300 μL using a 3 kD cut-off centrifuge column (Millipore, UFC900308). The protein concentration of the venom was quantified using a Bradford assay (Bio-Rad, 500–0006).

### Protein gel electrophoresis and silver staining

*S*. *feltiae* ESPs were prepared for gel electrophoresis by boiling for 5–10 minutes in 1x Laemmli sample buffer supplemented with 50mM Dithiothreitol (DTT) (Bio-Rad, 1610747). The denatured proteins were loaded into a Mini-PROTEAN TGX precast gels (Bio-Rad, 4561086) and electrophoresed at 100 V for 60–90 minutes. Silver staining was done following the manufacturer’s protocol (Pierce, # 24600).

### Testing *S*. *feltiae* IJ venom toxicity

*S*. *feltiae* ESPs toxicity was tested *in vivo* on *Drosophila melanogaster* flies as previously described [5, 83]. Adult male flies 5–6 days old were anesthetized with CO2 and injected with 20 ng of ESPs in a volume of 50 nl using pulled glass needles and a highspeed pneumatic microinjector (Tritech Research, MINJ-FLY). PBS was injected as a negative control. After injection the flies were transferred to new vials containing food and stored at 25°C with 60% relative humidity on a 12hr light/dark cycle. Survival of the flies was recorded over 40 days or until all the flies had died. ESP collection and toxicity testing were done in 3 biological replicates for each time point (PBS, 0 hr, 6 hr, 12 hr, 18 hr, 24 hr, 34 hr) with 3 technical replicates of each biological replicate. At least 60 flies were used for each technical replicate totaling at least 180 flies for each biological replicate.

### Vital staining for nematode damage assay

Nematodes were activated *in vitro* as described in the “Activation of IJs” section of the methods however scaled down to fit a 9 cm petri dish (0.082 g of sponge, 1 mL of 25% insect homogenate, and approximately 25,000 *S*. *feltiae* IJs). The sponge pieces were each pressed down 5 times before the nematodes were washed out and rinsed with 4 rounds of autoclaved PBS. The nematodes were then stained by mixing an equal volume of nematodes with an equal volume of 0.4% trypan blue (Sigma-Aldrich) to give a final dye concentration of 0.2%. The mixture was allowed to sit for 5 minutes before transferring the nematodes to a microscope slide for viewing and counting. This was replicated 3 times for each time point (6, 12, 18, 24, 30 hours of activation) with approximately 5000 counts each replicate (15,000 total counts for each time point) Representative images are in S2 Fig and raw counts in S2 Table.

### Axenic nematode production and assay

Axenic nematode production and assaying was done as previously described [5] with some slight modifications. Axenic *S*. *feltiae* IJs were produced *in vitro* by growing bleach sterilized *S*. *feltiae* eggs on the colonizing defective mutant bacterial strain of *Xenorhabdus nematophila*, *HGB315* [84]. HGB315 is unable to colonize the nematodes however can still be a food source. Phase I of the HGB315 bacteria colonies (blue) were obtained and verified using NBTA agar plates (40 mg/L 2,3,5-triphenyltetrazolium, 25 mg/L bromothymol blue, 8 g/L nutrient agar, supplemented with 0.1% (w/v) sodium pyruvate) and double checked with MacConkey Agar plates (reddish brown) (Difco MacConkey Agar, #212123, supplemented with 0.1% (w/v) sodium pyruvate). HGB315 was cultured in LB broth supplemented with 0.1% (w/v) sodium pyruvate over night at 28°C and shaking at 220 rpm.100–150 μl of overnight HGB315 liquid culture was spread on lipid agar plates (4 ml/L corn oil, 7 ml/L of corn syrup, 5 g/L of yeast extract, 2 g/L MgCl_2_, 8 g/L of nutrient broth,15 g/L of Bacto Agar, supplemented with 0.1% (w/v) sodium pyruvate) and incubated at 28°C overnight to form a thin layer of bacterial lawn. Surface sterilized *S*. *feltiae* eggs in a minimal volume of sterile Ringer’s solution (172 mM KCl, 68 mM NaCl, 5 mM NaHCO3, pH 6.1) was dropped onto the lipid agar plates and allowed to develop into gravid females. This is the first round pass to produce F1 generations of *S*. *feltiae* nematodes that were exposed only to the non-colonizing HGB315. HGB315 is a strain of *X*. *nematophila* which is not the native symbiotic bacteria of *S*. *feltiae* (*Xenorhabdus bovienii*), therefore these nematodes develop and become gravid much slower at approximately 5–6 days (versus ~4 days on *X*. *bovienii*) post seeding. To obtain axenic eggs, gravid females were rinsed in autoclaved 0.8% NaCl solution for 3 times followed with rocking in axenizing solution (0.7% NaOCl (bleach)/0.5 M NaOH) for 7.5 minutes for 3 times. Brief vortexing was applied 2–3 times in the first two rounds of axenizing to ensure mixing and degradation of adult nematode tissue. After the axenizing treatment, the eggs were rinsed in autoclaved Ringer’s solution for 3 times followed by incubation in a triple antibiotic solution (Penicillin, Neomycin, Streptomycin) for 30–45 minutes. The eggs were then rinsed with autoclaved Ringer’s solution for 3 times and centrifuged at 700 rcf for 1 min and the supernatant was removed to create a highly dense egg suspension with minimal liquid volume. Approximately 500,000 eggs were gently dispersed onto the lipid agar plates containing the HGB315 bacteria. When the bacteria were depleted, the nematodes were washed off and split into 3–5 new HGB315 bacteria plates. The *S*. *feltiae* nematodes were kept on the plates until they reached a high density and IJs can be seen crawling up the sides of the plates. At this point the population was still a mix of different life stages so the nematodes were transferred to White traps to collect axenic IJs.

### Axenic assay

To assay for non-colonization of bacteria inside *S*. *feltiae* IJs: approximately 1000 IJs were rinsed 3 times with autoclaved Ringer’s solution, followed by surface sterilization with 4 mM Hyamine 1622 solution (Sigma, 51126) for 30 minutes, and rinsed 3 times with Ringer’s solution. The IJs were then concentrated to a volume of 50 μl and homogenized with a tissue grinder (Fisher Scientific, 12-141-363). The homogenate was then plated onto LB plates (supplemented with 0.1% (w/v) sodium pyruvate) and incubated at 28°C in the dark. The plates were checked for bacterial growth for 5 days (S3 Fig). This was replicated 3 times for each batch of axenic *S*. *feltiae* IJs.

### Mass spectrometry of *S*. *feltiae* ESPs

To prepare *S*. *feltiae* ESPs for mass spectrometry analysis, the proteins were first precipitated with 80% acetone (-20°C pre-chilled) at 4:1 acetone to sample volume. The mixture was vortexed for 5 seconds 3 times and stored at -20°C overnight. The mixture was then centrifuged at 15,000 rcf for 10 minutes at 4°C to pellet the precipitated proteins. The supernatant was carefully removed, followed by addition of fresh -20°C chilled 80% acetone, and mixing by pipetting. The mixture was then centrifuged at 15,000 rcf for another 10 minutes. This process was repeated one more time and after removal of the 2^nd^ 80% acetone wash the protein pellet was allowed to air dry for 5 minutes. The protein pellet was then digested using the Trypsin/Lys-C, Mass Spec Grade kit (Promega, V5071) following the manufacturer’s Two-Step In-Solution Digestion protocol. Briefly, the protein pellet was suspended in 7 M urea/50 mM Tris-HCl (pH 8), followed by addition of DTT to a final concentration of 5 mM, and incubated at 37°C for 30 minutes. Iodoacetamide was then added to a final concentration of 15 mM, and incubated at room temperature for 30 minutes in the dark. The Trypsin/Lys-C protease mix was added at a ratio of 25:1 (protein: protease (w/w)) and incubated at 37°C for 4 hours. The mixture was then diluted with 50 mM Tris-HCl (pH 8) to reduce the urea concentration to approximately 0.5 M and continued incubation at 37°C overnight. Trifluoroacetic acid (TFA) was added to a final concentration of 0.5–1% to terminate digestion and the mixture was centrifuged at 15,000 rcf for 10 minutes to pellet particulate matter. The supernatant containing digested protein was cleaned using a C18 spin column (Pierce, 89873) following the manufacturer’s protocol.

### Mass spectrometry

Online 2D-nano LC/MS/MS was used to perform MudPIT mass spec analysis of *S*. *feltiae* ESPs. The mass spec apparatus consisted of a 2D nanoAcquity UPLC (Waters, Milford, MA) configured with an Orbitrap Fusion MS (Thermo Scientific, San Jose, CA). LC solutions/fractionation and MS parameters were as previously described [5]. The raw mass spec data was processed/analyzed with the Proteome Discoverer 2.2 software (Thermo Scientific, San Jose, CA) with the Sequest HT search engine running against the *S*. *feltiae* protein profile, steinernema_feltiae.PRJNA204661.WBPS11.protein.fa (Parasite.Wormbase.org). Duplicate genes were removed and only genes with FDR <5% were considered for further analysis. The raw mass spec data have been uploaded to the ProteomeXchange repository and can be accessed with the following links.

0 hr: **ftp://massive.ucsd.edu/MSV000082993**

6 hr: **ftp://massive.ucsd.edu/MSV000082997**

### Protein domain and peptidase analyses

Protein/peptide sequences of *S*. *feltiae* ESPs obtained from mass spec and the protein domain families were analyzed using the Pfam database and the hmmscan program (E-value < 10^−5^) of the HMMER software 3.0 as described [85]. Peptidase types based on the catalytic center amino acid (Serine, Metallo, Aspartic, etc.) and peptidase inhibitors were identified by BLAST+ against the MEROPS Peptidase database [86] from https://www.ebi.ac.uk/Tools/sss/ncbiblast/. Only hits with an E-value of <10^−5^ were further analyzed.

### Single nematode transcriptome sequencing

*S*.*feltiae* single nematode transcriptome sequencing was done as previously described [5, 6]. *In vitro* activated IJs were activated as described in the Activation of IJs section of the methods but scaled down to fit in a 6 cm petri dish with 1 ml of insect homogenate, 0.08 g of sponge, and 25,000 IJs [16, 17]. The IJs were activated for time points 3, 6, and 9 hrs. After activation the IJs were washed out of the sponge with autoclaved 0.8% NaCl and transferred to 1.5 ml eppendorf tubes. The IJs were cleaned by spinning down and removing/replacing the NaCl supernatant 4 times. We used only IJs that displayed fully activated morphology (confirmed by microscope) for each time point. This method, though arguably not highly representative of the entire population, was used in order to consistently select for individuals that were activating the fastest for each time point and minimize variation from nematodes with different levels of activation. The IJs were then transferred to RNase-free water before lysis. Naïve (0 hr) IJs were not exposed to any insect tissue and washed before proceeding to lysis. *In vivo* activated *S*. *feltiae* IJs were activated by infecting live waxworms at 50 IJs/waxworm. After 30 minutes the waxworms were gently rinsed in autoclaved 0.8% NaCl to wash off IJs that were on the surface of the waxworms but had not entered the waxworm. The infected waxworms were then stored in the dark at 25°C with 60% relative humidity for 3, 6, 9, 12, or 15 hrs. After the specified hours, the waxworms were individually placed in 6 cm petri dishes with autoclaved 0.8% NaCl and the activated IJs were dissected out. The IJs were washed by transferring them to new 6 cm petri dishes with fresh autoclaved NaCl 5x until being transferred to RNase free water before lysis. Activated IJs for each time point/condition (6 hr *in vitro*, 12 hr *in vivo*, etc.) were individually isolated in RNase-free water, cut into 3–4 pieces, and immediately transferred to lysis buffer containing RNAse inhibitor Proteinase K. The sample was placed on ice and observed periodically until the nematode tissue had been digested (typically 45–60 minutes). The sample was then incubated in a thermocycler at 85°C for 3 minutes to deactivate proteinase K. dNTP/ Oligo-dT_30_VN (5′-AAGCAGTGGTATCAACGCAGAGTACT30VN-3′) was added to the sample and poly-A RNA was reverse transcribed in a reaction solution of 100 U Superscript II RT (Thermo Fisher Scientific, 18064014), 10 U RNase inhibitor (Promega, N2611), 1x Superscript II first-strand buffer, 5 mM DTT, 1 M Betaine, 6 mM MgCl2, 1 μM TSO (LNA-modified TSO 5′-AAGCAGTGGTATCAACGCAGAGTACATrGrG+G-3′, Exiqon.com), and RNase-free water. The reverse transcription program was set to 1) 42°C 90 min, 2) 50°C 2 min, 42°C 2 min (repeat 14x), 3) 70°C 15 min, and 4) 4°C Hold. The cDNA was then added to a cDNA amplification mix with final concentrations of 1x KAPA HiFi HotStart ReadyMix (Kapa Biosystems, KK2602), 0.1 μM IS PCR primer (5′-AAGCAGTGGTATCAACGCAGAGT-3′, ordered from idtdna.com), and RNase-free water. The cDNA amplification program was set to 1) 98°C 3 min, 2) 98°C 20 sec, 67°C 15 sec, 72°C 6 min (repeat 17x), 3) 72°C 5 min, and 4) 4°C Hold. To clean the amplified cDNA, it was mixed with Ampure XP beads at a ratio of 1:1 (v/v). The mixture was then placed on a magnetic bead stand to magnetize the cDNA-bound beads to side-wall of the tube and washed with 3 rounds of 80% ethanol. After removal of the final ethanol wash the beads were air dried for 3–4 minutes and observed frequently under a microscope. At the first sign of a dry crack in the beads, 17.5 μl of elution Buffer (EB, 10 mM Tris-Cl, pH 8.5) was added, and incubated for 2 minutes. The sample was placed back on the magnetic bead stand for 2–3 minutes to separate the beads from the EB solution (now containing clean cDNA) and the EB solution was collected. cDNA concentration was measured by Qubit Fluorometer (Thermo Fisher Scientific) and the quality was analyzed by BioAnalyzer (Agilent).

The cDNA was tagmented using the Nextera DNA library prep kit (Illumina, FC-121-1030) following the protocol in L. Serra, et al 2018. Briefly, 20 ng of cDNA in 8 μl was mixed with 10 μl of Tagment DNA buffer and 2.2 μl of Tagment DNA enzyme from the kit. The mixture was incubated at 55°C for 5 minutes and cleaned up using the QIAquick DNA cleanup column (QIAGEN, 28104). The tagmented cDNA was then amplified using the Phusion High Fidelity PCR master mix (New England Biolabs, M0531L) with 30 μl of tagmented cDNA, 2.5 μl of Primer-1 (Ad1_no MX), 2.5 μl of Primer-2(Ad2.#), and 35 μl of Phusion High Fidelity PCR master mix buffer. The amplification program was set to 1) 72°C 5 min, 2) 98°C 30 sec, 3) 98°C 10 sec, 63°C 30 sec., 72°C 1 min (repeat 10x), and 4) 4°C Hold. The sample was then cleaned up with Ampure XP beads as described above except, scaling up to use 30 μl of EB and collecting 27.5 μl of the supernatant. Libraries were prepared and sequenced as paired-end, 43 base pair reads on the Illumina Nextseq 500.

### Gene expression quantification

Unstranded, paired-end 43 bp RNA-seq reads for each worm were mapped to the *S*. *feltiae* transcriptome downloaded from WormBase ParaSite (WS263) using Bowtie 1.0.0 with the following options: -X 1500 -a -m 200—S—seedlen 25 -n 2—offrate 1 -p 64 -v 3 [87]. After Bowtie, gene expression was quantified with RSEM with the following options: rsem-calculate-expression—bam—paired-end. Gene expression for *S*. *carpocapsae* were performed as previously described [5] and reported in Transcripts Per Million (TPM). We used counts for differential gene expression analysis. Reads for single worm RNA-seq samples were submitted to Gene Expression Omnibus (GEO) under the accession number GSE119223.

### Normalization and batch correction

The Transcript per million (TPM) generated by rsem-calculate-expression for *S*. *feltiae* samples were normalized according to groups using the R package limma [88] because samples were collected, processed and sequenced in different batches. Samples were batched corrected between 3 and 9 hours *in vitro* to 6 hours *in vitro*, 3,6,9,12,15 hours *in vivo* with edgeR package removebatcheffects with log2 of TPM matrix. Normalization and batch correction for *S*. *carpocapsae* were done as previously described [5].

### MRNA and protein correlation

Log_2_ of the average TPM+1 (transcripts per million, RNA levels) and Log_2_ of the emPAI (protein abundance levels) for the 266 genes of *S*. *feltiae* ESPs was plotted in Rstudio using the package ggplot2 [89]. Pearson’s correlation and Spearman’s rank correlation were calculated using Excel.

### Gene expression analysis and GO enrichment analysis

Differential gene expression was determined using edgeR [32]. Counts were normalized by library size using calcNormFactors. Genes were called differentially expressed if FDR < 0.05 and fold change > 2. The list of genes that were differentially expressed (DE) using edgeR were used to create a TPM matrix.

Gene expression in TPM were clustered using Cluster 3.0 [90] with the following options: log transformed, mean centered, normalized. Then genes were hierarchically clustered with center correlation. Heatmap were visualized with Java TreeView [91]. Heatmap for Fig 5C were done using the R package heatmap.2 with centroid hierarchical clustering by row.

MaSigPro was run as a two-time series to evaluate the differences and similarities of gene expression between *in vitro* and *in vivo* time course with 5670 differentially expressed genes found with edgeR between 6 hours *in vitro* activated and naïve IJs. Gene ontology enrichment analyses was calculated using Blast2GO Fisher’s exact test and considered statistically significant if FDR < 0.05 [92]. List of genes used in Blast2GO were differentially expressed according to edgeR or dynamically expressed according to maSigPro.

### Venom orthology analysis

We obtained a list of N:N orthologs and paralogs between *S*. *feltiae* and *S*. *carpocapsae* from WormBase ParaSite Biomart. List were obtained by choosing *S*. *feltiae* genome as query to find orthologs and paralogs in *S*. *carpocapsae*. List of venom proteins for *S*. *carpocapsae* were obtained from Lu et al. 2017 and compared to list of *S*. *feltiae* venom proteins. Orthology analysis was done with edgeR with function “match”. In determining the orthology of *S*. *feltiae* L889_g32029 (*Sf-flp-21*) to *C*. *elegans flp-21*, we relied on the predicted sequence of the mature peptide [34, 93]. Using this method, we determined that, similar to *Sc-flp-21*, *Sf-flp-21* has an identical predicted mature peptide as the *flp-21* from *C*. *elegans*.

## Supporting information

S1 Fig Average concentrations of ESPs collected from different batches of *S. feltiae* IJs activated over time. All batches were activated the same way as described in the IJ activation section of the methods and a final volume of 300 μl was collected for each time point. Each time point was repeated 3 times and the protein concentrations were determined by a Bradford assay.
(PDF)

S2 Fig Damaged nematodes from sponge activations. Representative pictures of damaged (indicated by trypan blue staining, 0.2% final concentration) and undamaged nematodes at various time points (A) 6 hours, (B) 18 hours, (C) 24 hours, (D) 30 hours of activation. Image B shows a view of undamaged nematodes at 18 hours while the rest show instances of damaged nematodes. (E) Percentages of the nematode population that exhibited stained damaged tissue from (uncrushed) normal sponge activation experiments. (F) Percentages of the nematode population that exhibited stained damaged tissue from manual crushing of sponge activations (pink) combined with data from panel E (blue). Bars represent the mean of 3 biological replicates with 5000 counts each and error bars represent standard deviation. **** represent statistical significance with P<0.0001. Statistical analysis was done using Graphpad Prism 8.0 software running unpaired one-way ANOVA with (recommended) Dunnett’s multiple comparisons test. The raw data counts can be found in S2 Table.
(PDF)

S3 Fig Axenic *S. feltiae* Assay, ESP, Activity. A) Schematic of how IJs were plated to assay for axenic IJs. A1) Grounded bleach surface sterilized *S*. *feltiae* IJs (symbiotic or axenic) on an NBTA plate supplemented with sodium pyruvate. Blue colonies on NBTA plates represent primary phase *X*. *bovenii*. A2) Grounded Hyamine surface sterilized *S*. *feltiae* IJs on an NBTA plate supplemented with sodium pyruvate. A3) Grounded bleach surface sterilized *S*. *feltiae* IJs (symbiotic or axenic) on an LB plate supplemented with sodium pyruvate (SP). A4) Grounded Hyamine surface sterilized *S*. *feltiae* IJs on an LB plate supplemented with sodium pyruvate. This was repeated 3 times using approximately 1000 IJs for each batch of *S*. *feltiae* IJs.
B) Silver stained protein gel of ESPs collected from symbiotic (S) and axenic (A) *S*. *feltiae* IJs activated for 6 hours. C) Survival curve of *D*. *melanogaster* fruit flies injected with 20 ng of ESPs collected from axenic *S*. *feltiae* IJs activated for 6 hrs. This was repeated 3 times with at least 90 flies for reach replicate.
(PDF)

S4 Fig Genes differentially expressed during *in vivo* IJ activation. (A) maSigPro profiles of genes clusters during *in vivo* time course activation. (B) Representative GO terms for each maSigPro cluster. (C) heatmap of neuropeptide pathway enriched genes from cluster 2.
(PDF)

S5 Fig mRNA-Protein Correlation of *S. feltiae* ESPs. Correlation plot of mRNA abundance (log2 of TPM+1) to protein abundance (log2 of emPAI).
(PDF)

S6 Fig Core venom orthologs in non-*Steinernema* organisms. Pie chart of the 52 core ESPs which had orthologs in genera other than Steinernema and categorized into either vertebrate-parasitic nematodes, non-parasitic nematodes, or non-nematodes. The list of best orthologs found in non-Steinernema organisms can be found in S4 Table, which was produced using Blast2Go blastp default settings (E-value <1x10-3).
(PDF)

S1 Table *S. feltiae* IJ time course activation rates and statistical comparison to *S. carpocapsae* rates. 1A) Table with the counts of *S*. *feltiae* IJs that were either fully activated, partially activated, or non-activated. Activation rates were quantified for each time point 3 times. The average percent of activation was calculated with standard error of the mean (SEM) and standard deviation (SD) shown below. The activation rate data for naïve/0-hour *S*. *carpocapsae* is also included as this data was obtained in this study. P-values from paired two-way ANOVA with (Prism recommended) Sidak’s multiple comparisons test comparing *S*. *feltiae* activation time points/categories relative to *S*. *carpocapsae* (*S*. *carpocapsae* activation rates used in statistical analyses (except naïve/0 hour) are not shown and were obtained from Lu et al, 2017[5]).
(XLSX)

S2 Table Damaged nematode count data. Raw data assessing the number of damaged nematodes shown in S2 Fig.
(XLSX)

S3 Table ES proteins from *S. feltiae* 6 hr and 0 hr symbiotic. Table of ESPs identified by mass spec from naïve (0 hr) or 6 hr activated *S*. *feltiae* IJs used in our analyses. Duplicate genes were removed and only genes with FDR<5% are included in these lists. This filter resulted in 266 total proteins from 6 hr activated IJs and 682 total proteins from naïve IJs. The raw mass spec data (which includes proteins not used in our analyses) have been uploaded to the ProteomeXchange repository and can be accessed with the following links.
0 hr: **ftp://massive.ucsd.edu/MSV000082997**.
6 hr: **ftp://massive.ucsd.edu/MSV000082997**.
(XLSX)

S4 Table Core venom proteins. List of 52 core venom protein gene IDs shared between *S*. *feltiae* (L889) and *S*. *carpocapsae* (L596) as well as their associated blast descriptions.
(XLSX)

S5 Table 112 *S. feltiae* venom orthologs to *S. carpocapsae*. List of 112 *S*. *feltiae* (L889) venom gene IDs with homologs in *S*. *carpocapsae* (L596) venom [5].
(XLSX)

S6 Table 183 *S. carpocapsae* venom orthologs to *S. feltiae*. List of 183 *S*. *carpocapsae* (L596) venom gene IDs [5] with homologs in *S*. *feltiae* (L889) venom.
(XLSX)

S7 Table Core venom GO terms. List of enriched GO terms associated with the 52 core venom proteins between *S*. *feltiae* and *S*. *carpocapsae*.
(XLSX)

## References

[1] Hotez PJ, Alvarado M, Basanez MG, Bolliger I, Bourne R, Boussinesq M, et al The global burden of disease study 2010: interpretation and implications for the neglected tropical diseases. PLoS Negl Trop Dis. 2014;8(7):e2865 10.1371/journal.pntd.0002865 25058013 PMC4109880

[2] Pullan RL, Smith JL, Jasrasaria R, Brooker SJ. Global numbers of infection and disease burden of soil transmitted helminth infections in 2010. Parasites & Vectors. 2014;7:19 10.1186/1756-3305-7-37 WOS:000334641400001. 24447578 PMC3905661

[3] Cuesta-Astroz Y, de Oliveira FS, Nahum LA, Oliveira G. Helminth secretomes reflect different lifestyles and parasitized hosts. International Journal for Parasitology. 2017;47(9):529–44. 10.1016/j.ijpara.2017.01.007 WOS:000408076200003. 28336271

[4] Stoltzfus JD, Pilgrim AA, Herbert DR. Perusal of parasitic nematode 'omits in the post-genomic era. Molecular and Biochemical Parasitology. 2017;215:11–22. 10.1016/j.molbiopara.2016.11.003 WOS:000405049100003. 27887974 PMC5440216

[5] Lu DH, Macchietto M, Chang D, Barros MM, Baldwin J, Mortazavi A, et al Activated entomopathogenic nematode infective juveniles release lethal venom proteins. Plos Pathogens. 2017;13(4):31 10.1371/journal.ppat.1006302 WOS:000402555700018. 28426766 PMC5398726

[6] Serra L, Chang D, Macchietto M, Williams K, Murad R, Lu D, et al Adapting the Smart-seq2 Protocol for Robust Single Worm RNA-seq. Bio-protocol. 2018;8(4):e2729 10.21769/BioProtoc.2729 29564372 PMC5857950

[7] Cassada RC, Russell RL. The dauer larva, a post-embryonic developmental variant of the nematode *Caenorhabditis elegans*. Developmental Biology. 1975;46(2):326–42. 10.1016/0012-1606(75)90109-8. 1183723

[8] Hallem EA, Dillman AR, Hong AV, Zhang Y, Yano JM, DeMarco SF, et al A sensory code for host seeking in parasitic nematodes. Curr Biol. 2011;21(5):377–83. 10.1016/j.cub.2011.01.048 21353558 PMC3152378

[9] Dillman AR, Guillermin ML, Lee JH, Kim B, Sternberg PW, Hallem EA. Olfaction shapes host–parasite interactions in parasitic nematodes. 2012 10.1073/pnas.1211436109 22851767 PMC3435218

[10] Kaya HK, Gaugler R. Entomophathogenic Nematodes. Annual Review of Entomology. 1993;38:181–206. 10.1146/annurev.en.38.010193.001145 WOS:A1993KF69700009.

[11] Crook M. The dauer hypothesis and the evolution of parasitism: 20 years on and still going strong. Int J Parasit. 2014;44(1):1–8. 10.1016/j.ijpara.2013.08.004 WOS:000331415400001. 24095839 PMC3947200

[12] Hawdon JM, Jones BF, Perregaux MA, Hotez PJ. *Ancylostoma*-*Caninum*—Metalloprotease Release Coincides with Activation of Infective Larvae in-Vitro. Experimental Parasitology. 1995;80(2):205–11. 10.1006/expr.1995.1025 ISI:A1995QP93400005. 7895832

[13] Campos-Herrera R, Barbercheck M, Hoy CW, Stock SP. Entomopathogenic Nematodes as a Model System for Advancing the Frontiers of Ecology. Journal of Nematology. 2012;44(2):162–76. WOS:000320451700010. 23482825 PMC3578465

[14] Hodson AK, Siegel JP, Lewis EE. Ecological influence of the entomopathogenic nematode, *Steinernema carpocapsae*, on pistachio orchard soil arthropods. Pedobiologia. 2012;55(1):51–8. 10.1016/j.pedobi.2011.10.005 WOS:000300211100007.

[15] Lewis EE, Campbell J, Griffin C, Kaya H, Peters A. Behavioral ecology of entomopathogenic nematodes. Biological Control. 2006;38(1):66–79. 10.1016/j.biocontrol.2005.11.007 WOS:000238596600006.

[16] Alonso V, Nasrolahi S, Dillman A. Host-Specific Activation of Entomopathogenic Nematode Infective Juveniles. Insects. 2018;9(2):59 10.3390/insects9020059 29865224 PMC6023527

[17] Lu DH, Sepulveda C, Dillman AR. Infective Juveniles of the Entomopathogenic Nematode *Steinernema scapterisci* Are Preferentially Activated by Cricket Tissue. Plos One. 2017;12(1):14 10.1371/journal.pone.0169410 WOS:000391612300184. 28046065 PMC5207650

[18] Balasubramanian N, Hao YJ, Toubarro D, Nascimento G, Simoes N. Purification, biochemical and molecular analysis of a chymotrypsin protease with prophenoloxidase suppression activity from the entomopathogenic nematode *Steinernema carpocapsae*. Int J Parasit. 2009;39(9):975–84. 10.1016/j.ijpara.2009.01.012 ISI:000267569000004. 19249304

[19] Toubarro D, Avila MM, Montiel R, Simoes N. A Pathogenic Nematode Targets Recognition Proteins to Avoid Insect Defenses. Plos One. 2013;8(9):13 10.1371/journal.pone.0075691 WOS:000325423500086. 24098715 PMC3787073

[20] Lewis EE, Clarke DJ. Nematode Parasites and Entomopathogens In: Vega FE, Kaya HK, editors. Insect Pathology, 2nd Edition San Diego: Elsevier Academic Press Inc; 2012 p. 395–424.

[21] Hunt DJ, Nguyen KB, Spiridonov SE. Steinernematidae: species descriptions Advances in entomopathogenic nematode taxonomy and phylogeny: Brill; 2016 p. 111.

[22] Poinar GO Jr. Nematodes for biological control of insects. 1979.

[23] Hodson AK, Friedman ML, Wu LN, Lewis EE. European earwig (*Forficula auricularia*) as a novel host for the entomopathogenic nematode *Steinernema carpocapsae*. Journal of Invertebrate Pathology. 2011;107(1):60–4. 10.1016/j.jip.2011.02.004 WOS:000289831300008. 21356215

[24] Nguyen KB, Smart GC. *Steinernema scapterisci* n. sp. (Rhabditida, Steinernematidae). Journal of Nematology. 1990;22(2):187–99. WOS:A1990CY91100007. 19287709 PMC2619029

[25] Stock SP, Koppenhofer AM. *Steinernema scarabaei* n. sp (Rhabditida: Steinernematidae), a natural pathogen of scarab beetle larvae (Coleoptera: Scarabaeidae) from New Jersey, USA. Nematology. 2003;5:191–204. 10.1163/156854103767139680 WOS:000184809500004.

[26] Adams BJ, Peat SM, Dillman AR. Phylogeny and evolution In: Nguyen KB, Hunt DJ, editors. Entomopathogenic nematodes: Systematics, phylogeny, and bacterial symbionts. Nematology monographs and perspectives. 5 Leiden-Boston: Brill; 2007 p. 693–733.

[27] Spiridonov SE, Reid AP, Podrucka K, Subbotin SA, Moens M. Phylogenetic relationships within the genus *Steinernema* (Nematoda: Rhabditida) as inferred from analyses of sequences of the ITSI-5.8S-ITS2 region of rDNA and morphological features. Nematology. 2004;6:547–66. 10.1163/1568541042665304 ISI:000226415300008.

[28] Dunphy GB, Webster JM. Partially characterized components of the epicuticle of dauer juveniles *Steinernema feltiae* and their influence on hemocyte activity in *Galleria mellonella*. Journal of Parasitology. 1987;73(3):584–8. 10.2307/3282140 WOS:A1987J343300020.

[29] Brivio MF, Pagani M, Restelli S. Immune suppression of *Galleria mellonella* (Insecta, Lepidoptera) humoral defenses induced by *Steinernema feltiae* (Nematoda, Rhabditida): involvement of the parasite cuticle. Experimental Parasitology. 2002;101(2–3):149–56. 10.1016/s0014-4894(02)00111-x WOS:000179436600009. 12427469

[30] Brivio MF, Mastore M, Moro M. The role of *Steinernema feltiae* body-surface lipids in host-parasite immunological interactions. Molecular and Biochemical Parasitology. 2004;135(1):111–21. 10.1016/j.molbiopara.2004.01.012 WOS:000221489500012. 15287592

[31] Brivio M, Mastore M. Nematobacterial Complexes and Insect Hosts: Different Weapons for the Same War. Insects. 2018;9(3):117 10.3390/insects9030117 30208626 PMC6164499

[32] Robinson MD, McCarthy DJ, Smyth GK. edgeR: a Bioconductor package for differential expression analysis of digital gene expression data. Bioinformatics. 2010;26(1):139–40. 10.1093/bioinformatics/btp616 19910308 PMC2796818

[33] Conesa CA, Nueda J. maSigPro: Significant Gene Expression Profile Differences in Time Course Gene Expression Data. 1.52.0 ed: R package; 2018.

[34] Morris R, Wilson L, Sturrock M, Warnock ND, Carrizo D, Cox D, et al A neuropeptide modulates sensory perception in the entomopathogenic nematode *Steinernema carpocapsae*. Plos Pathogens. 2017;13(3):17 10.1371/journal.ppat.1006185 WOS:000398120300047. 28253355 PMC5333901

[35] Finn RD, Coggill P, Eberhardt RY, Eddy SR, Mistry J, Mitchell AL, et al The Pfam protein families database: towards a more sustainable future. Nucleic Acids Research. 2016;44(D1):D279–D85. 10.1093/nar/gkv1344 WOS:000371261700038. 26673716 PMC4702930

[36] Hu PJ. Dauer. WormBook 2007 10.1895/wormbook.1.144.1 PMC2890228 17988074

[37] Viney ME, Lok JB. The biology of *Strongyloides* spp. WormBook 2015 10.1895/wormbook.1.141.2 PMC5402216 26183912

[38] Bonner TP. Initiation of Development *In vitro* of Third-Stage *Nippostrongylus brasiliensis*. The Journal of Parasitology. 1979;65(1):74–8. 10.2307/3280205 448602

[39] Hawdon JM, Volk SW, Pritchard DI, Schad GA. Resumption of Feeding *Invitro* by Hookworm Third-Stage Larvae—a Comparative Study. Journal of Parasitology. 1992;78(6):1036–40. 10.2307/3283226 ISI:A1992KL32200013. 1491295

[40] Dillman AR, Macchietto M, Porter CF, Rogers A, Williams B, Antoshechkin I, et al Comparative genomics of *Steinernema* reveals deeply conserved gene regulatory networks. Genome Biol. 2015;16:200 10.1186/s13059-015-0746-6 26392177 PMC4578762

[41] Adams BJ, Peat SM, Dillman AR. Phylogeny and evolution. Entomopathogenic Nematodes: Systematics, Phylogeny and Bacterial Symbionts. 2007;5:693–733. ISI:000301864200007.

[42] Hominick WM, Reid AP. Perspectives on entomopathogenic nematology. Boca Raton, FL 33431: CRC Press Inc; 1990 p. 327–45.

[43] Griffin CT. Effects of prior storage conditions on the infectivity of *Heterorhabditis* sp (Nematoda: Heterorhabditidae). Fundamental and Applied Nematology. 1996;19(1):95–102. ISI:A1996TP25100014.

[44] Campbell JF, Koppenhofer AM, Kaya HK, Chinnasri B. Are there temporarily non-infectious dauer stages in entomopathogenic nematode populations: a test of the phased infectivity hypothesis. Parasitology. 1999;118 (Pt 5):499–508. . 10363283 10.1017/s0031182099003984

[45] Dempsey CM, Griffin CT. Phased activity in *Heterorhabditis megidis* infective juveniles. Parasitology. 2002;124:605–13. 10.1017/S0031182002001609 ISI:000177212400005. 12118716

[46] Grewal PS, Lewis EE, Gaugler R. Response of infective stage parasites (Nematoda: Steinernematidae) to volatile cues from infected hosts. Journal of Chemical Ecology. 1997;23(2):503–15. 10.1023/B:Joec.0000006374.95624.7e ISI:A1997WR84600018.

[47] Hewitson JP, Grainger JR, Maizels RM. Helminth immunoregulation: The role of parasite secreted proteins in modulating host immunity. Molecular and Biochemical Parasitology. 2009;167(1):1–11. 10.1016/j.molbiopara.2009.04.008 ISI:000268049600001. 19406170 PMC2706953

[48] Lightowlers MW, Rickard MD. Excretory Secretory Products of Helminth-Parasites—Effects on Host Immune-Responses. Parasitology. 1988;96:S123–S66. ISI:A1988M840500009. 3287288 10.1017/s0031182000086017

[49] Holland MJ, Harcus YM, Riches PL, Maizels RM. Proteins secreted by the parasitic nematode Nippostrongylus brasiliensis act as adjuvants for Th2 responses. European Journal of Immunology. 2000;30(7):1977–87. 10.1002/1521-4141(200007)30:7<1977::AID-IMMU1977>3.0.CO;2-3 ISI:000088262300020. 10940887

[50] Healer J, Ashall F, Maizels RM. Characterization of Proteolytic Enzymes from Larval and Adult *Nippostrongylus brasiliensis*. Parasitology. 1991;103:305–14. 10.1017/S0031182000059588 ISI:A1991GH06800016. 1745556

[51] Hewitson JR, Harcus YM, Curwenb RS, Dowle AA, Atmadja AK, Ashton PD, et al The secretome of the filarial parasite, *Brugia malayi*: Proteomic profile of adult excretory-secretory products. Molecular and Biochemical Parasitology. 2008;160(1):8–21. 10.1016/j.molbiopara.2008.02.007 ISI:000257024600002. 18439691

[52] Dillman AR, Chaston JM, Adams BJ, Ciche TA, Goodrich-Blair H, Stock SP, et al An Entomopathogenic Nematode by Any Other Name. Plos Pathogens. 2012;8(3):4 10.1371/journal.ppat.1002527 WOS:000302225600003. 22396642 PMC3291613

[53] Aryal SK, Lu D, Le K, Allison L, Gerke C, Dillman AR. Sand crickets (*Gryllus firmus*) have low susceptibility to entomopathogenic nematodes and their pathogenic bacteria. J Invertebr Pathol. 2019;160:54–60. Epub 2018/12/12. 10.1016/j.jip.2018.12.002 . 30528638

[54] Han RC, Ehlers RU. Pathogenicity, development, and reproduction of *Heterorhabditis bacteriophora* and *Steinernema carpocapsae* under axenic *in vivo* conditions. Journal of Invertebrate Pathology. 2000;75(1):55–8. 10.1006/jipa.1999.4900 WOS:000085395100009. 10631058

[55] Poinar GO, Thomas GM. Significance of *Achromobacter nematophilus* Poinar and Thomas (Achromobacteraceae—Eubacteriales) in Development of Nematode DD-136 (*Neoaplectana* sp Steinernematidae). Parasitology. 1966;56:385–&. WOS:A19667764600015. 4960247 10.1017/s0031182000070980

[56] Sicard M, Le Brun N, Pages S, Godelle B, Boemare N, Moulia C. Effect of native *Xenorhabdus* on the fitness of their *Steinernema* hosts: Contrasting types of interaction. Parasitology Research. 2003;91(6):520–4. 10.1007/s00436-003-0998-z WOS:000187993700016. 14557877

[57] Burman M. *Neoaplectana carpocapsae*: Toxin production by axenic Insect Parasitic Nematodes. Nematologica. 1982;28(1):62–70. WOS:A1982PU84500006.

[58] Walter TN, Dunphy GB, Mandato CA. *Steinernema carpocapsae* DD136: Metabolites limit the non-self adhesion responses of haemocytes of two lepidopteran larvae, *Galleria mellonella* (*F*. *Pyralidae*) and *Malacosoma disstria* (*F*. *Lasiocampidae*). Experimental Parasitology. 2008;120(2):161–74. 10.1016/j.exppara.2008.07.001 WOS:000259659600006. 18656470

[59] Hao YJ, Montiel R, Nascimento G, Toubarro D, Simoes N. Identification and expression analysis of the *Steinernema carpocapsae* elastase-like serine protease gene during the parasitic stage. Exp Parasitol. 2009;122(1):51–60. 10.1016/j.exppara.2009.01.014 WOS:000265463600009. 19545520

[60] Jing YJ, Toubarro D, Hao YJ, Simoes N. Cloning, characterisation and heterologous expression of an astacin metalloprotease, Sc-AST, from the entomoparasitic nematode *Steinernema carpocapsae*. Molecular and Biochemical Parasitology. 2010;174(2):101–8. 10.1016/j.molbiopara.2010.07.004 WOS:000283697900002. 20670659

[61] Toubarro D, Avila MM, Hao YJ, Balasubramanian N, Jing YJ, Montiel R, et al A Serpin Released by an Entomopathogen Impairs Clot Formation in Insect Defense System. Plos One. 2013;8(7):12 10.1371/journal.pone.0069161 WOS:000322064300088. 23874900 PMC3712955

[62] Ehlers RU, Wulff A, Peters A. Pathogenicity of axenic Steinernema feltiae, Xenorhabdus bovienii, and the bacto-helminthic complex to larvae of Tipula oleracea (Diptera) and Galleria mellonella (Lepidoptera). Journal of Invertebrate Pathology. 1997;69(3):212–7. 10.1006/jipa.1996.4647 WOS:A1997WZ20900003. 9170346

[63] Dunphy GB, Webster JM. Influence of *Steinernema feltiae* (Filipjev) Wouts, Mracek, Gerdin and Bedding DD136 strain on the humoral and haemocytic responses of *Galleria mellonella* (L.) larvae to selected bacteria. Parasitology. 1985;91(2):369–80. Epub 2009/04/01. 10.1017/s0031182000057437

[64] Odaly JA, Cabrera Z. Immunization of hamsters with TLCK-killed parasites induces protection against *Leishmania* infection. Acta Tropica. 1986;43(3):225–36. WOS:A1986E140800004. 2877549

[65] Redmond DL, Knox DP. Protection studies in sheep using affinity-purified and recombinant cysteine proteinases of adult *Haemonchus contortus*. Vaccine. 2004;22(31–32):4252–61. 10.1016/j.vaccine.2004.04.028 WOS:000224758200016. 15474716

[66] Ruiz A, Molina JM, Gonzalez JF, Conde MM, Martin S, Hernandez YI. Immunoprotection in goats against *Haemonchus contortus* after immunization with cysteine protease enriched protein fractions. Veterinary Research. 2004;35(5):565–72. 10.1051/vetres:2004032 WOS:000224585800005. 15369659

[67] Knox D. Proteases in blood-feeding nematodes and their potential as vaccine candidates In: Robinson MW, Dalton JP, editors. Cysteine Proteases of Pathogenic Organisms. Advances in Experimental Medicine and Biology. 712 Berlin: Springer-Verlag Berlin; 2011 p. 155–76. 10.1007/978-1-4419-8414-2_10 21660664

[68] McKerrow JH, Caffrey C, Kelly B, Loke P, Sajid M. Proteases in parasitic diseases. Annual Review of Pathology-Mechanisms of Disease Annual Review of Pathology-Mechanisms of Disease. 1 Palo Alto: Annual Reviews; 2006 p. 497–536. 10.1146/annurev.pathol.1.110304.100151 18039124

[69] Todorova VK, Stoyanov DI. Partial characterization of serine proteinases secreted by adult *Trichinella spiralis*. Parasitology Research. 2000;86(8):684–7. 10.1007/pl00008552 WOS:000088738100013. 10952270

[70] Cwiklinski K, Meskill D, Robinson MW, Pozio E, Appleton JA, Connolly B. Cloning and analysis of a *Trichinella pseudospiralis* muscle larva secreted serine protease gene. Veterinary Parasitology. 2009;159(3–4):268–71. 10.1016/j.vetpar.2008.10.036 WOS:000264038400019. 19054614 PMC2673510

[71] Rees-Roberts D, Mullen LM, Gounaris K, Selkirk ME. Inactivation of the complement anaphylatoxin C5a by secreted products of parasitic nematodes. International Journal for Parasitology. 2010;40(5):527–32. 10.1016/j.ijpara.2009.10.006 WOS:000277107400004. 19874826 PMC2852653

[72] Zhao YM, Sun W, Zhang P, Chi H, Zhang MJ, Song CQ, et al Nematode sperm maturation triggered by protease involves sperm-secreted serine protease inhibitor (Serpin). Proceedings of the National Academy of Sciences of the United States of America. 2012;109(5):1542–7. 10.1073/pnas.1109912109 WOS:000299731400044. 22307610 PMC3277156

[73] Drake LJ, Bianco AE, Bundy DAP, Ashall F. Characterization of peptidases of adult *Trichuris muris*. Parasitology. 1994;109:623–30. 10.1017/s0031182000076502 WOS:A1994PV35900010. 7831097

[74] Hotez PJ, Cerami A. Secretion of a proteolytic anticoagulant by *Ancylostoma* hookworms. Journal of Experimental Medicine. 1983;157(5):1594–603. 10.1084/jem.157.5.1594 WOS:A1983QQ38700018. 6343546 PMC2186995

[75] Kennedy MW, Corsico B, Cooper A, Smith BO. The Unusual Lipid-binding Proteins of Nematodes: NPAs, nemFABPs and FARs In: Kennedy MW, Harnett W, editors. Parasitic Nematodes: Molecular Biology, Biochemistry and Immunology, 2nd Edition Wallingford: Cabi Publishing-C a B Int; 2013 p. 397–412.

[76] Iberkleid I, Vieira P, Engler JD, Firester K, Spiegel Y, Horowitz SB. Fatty Acid-and Retinol-Binding Protein, Mj-FAR-1 Induces Tomato Host Susceptibility to Root-Knot Nematodes. Plos One. 2013;8(5):14 10.1371/journal.pone.0064586 WOS:000320362700159. 23717636 PMC3661543

[77] Vieira P, Kamo K, Eisenback JD. Characterization and silencing of the fatty acid- and retinol-binding Pp-far-1 gene in *Pratylenchus penetrans*. Plant Pathology. 2017;66(7):1214–24. 10.1111/ppa.12664 ISI:000409374900017.

[78] Phani V, Shivakumara TN, Davies KG, Rao U. *Meloidogyne incognita* Fatty Acid- and Retinol- Binding Protein (Mi-FAR-1) Affects Nematode Infection of Plant Roots and the Attachment of *Pasteuria penetrans* Endospores. Frontiers in Microbiology. 2017;8 10.3389/fmicb.2017.00008 ISI:000414123500001. 29209280 PMC5701614

[79] Abreu RD, Penalva LO, Marcotte EM, Vogel C. Global signatures of protein and mRNA expression levels. Molecular Biosystems. 2009;5(12):1512–26. 10.1039/b908315d WOS:000271727600013. 20023718 PMC4089977

[80] Grun D, Kirchner M, Thierfelder N, Stoeckius M, Selbach M, Rajewsky N. Conservation of mRNA and Protein Expression during Development of *C*.*elegans*. Cell Reports. 2014;6(3):565–77. 10.1016/j.celrep.2014.01.001 WOS:000331168400014. 24462290

[81] Koussounadis A, Langdon SP, Um IH, Harrison DJ, Smith VA. Relationship between differentially expressed mRNA and mRNA-protein correlations in a xenograft model system. Scientific Reports. 2015;5:9 10.1038/srep10775 WOS:000356137300001. 26053859 PMC4459080

[82] White GF. A method for obtaining infective nematode larvae from cultures. Science. 1927;66(1709):302–3. 10.1126/science.66.1709.302-a . 17749713

[83] Pham LN, Dionne MS, Shirasu-Hiza M, Schneider DS. A specific primed immune response in *Drosophila* is dependent on phagocytes. Plos Pathogens. 2007;3(3):8 10.1371/journal.ppat.0030026 WOS:000248495200006. 17352533 PMC1817657

[84] Heungens K, Cowles CE, Goodrich-Blair H. Identification of *Xenorhabdus nematophila* genes required for mutualistic colonization of S*teinernema carpocapsae* nematodes. Molecular Microbiology. 2002;45(5):1337–53. 10.1046/j.1365-2958.2002.03100.x WOS:000177750400015. 12207701

[85] Finn RD, Clements J, Eddy SR. HMMER web server: interactive sequence similarity searching. Nucleic Acids Research. 2011;39:W29–W37. 10.1093/nar/gkr367 ISI:000292325300006. 21593126 PMC3125773

[86] Rawlings ND, Waller M, Barrett AJ, Bateman A. MEROPS: the database of proteolytic enzymes, their substrates and inhibitors. Nucleic Acids Research. 2014;42(D1):D503–D9. 10.1093/nar/gkt953 WOS:000331139800075. 24157837 PMC3964991

[87] Langmead B, Trapnell C, Pop M, Salzberg SL. Ultrafast and memory-efficient alignment of short DNA sequences to the human genome. Genome Biology. 2009;10(3):10 10.1186/gb-2009-10-3-r25 WOS:000266544500005. 19261174 PMC2690996

[88] Ritchie ME, Phipson B, Wu D, Hu YF, Law CW, Shi W, et al limma powers differential expression analyses for RNA-sequencing and microarray studies. Nucleic Acids Research. 2015;43(7):13 10.1093/nar/gkv007 WOS:000354722500005. 25605792 PMC4402510

[89] Wickham H. ggplot2: Elegant Graphics for Data Analysis. Ggplot2: Elegant Graphics for Data Analysis. 2009:1–212. 10.1007/978-0-387-98141-3 WOS:000269437100014.

[90] de Hoon MJL, Imoto S, Nolan J, Miyano S. Open source clustering software. Bioinformatics. 2004;20(9):1453–4. 10.1093/bioinformatics/bth078 WOS:000222125600013. 14871861

[91] Saldanha AJ. Java Treeview-extensible visualization of microarray data. Bioinformatics. 2004;20(17):3246–8. 10.1093/bioinformatics/bth349 WOS:000225361400039. 15180930

[92] Conesa A, Gotz S, Garcia-Gomez JM, Terol J, Talon M, Robles M. Blast2GO: a universal tool for annotation, visualization and analysis in functional genomics research. Bioinformatics. 2005;21(18):3674–6. 10.1093/bioinformatics/bti610 . 16081474

[93] Li C, Kim K. Family of FLP peptides in *Caenorhabditis elegans* and related nematodes. Frontiers in Endocrinology. 2014;5 10.3389/fendo.2014.00150 WOS:000209749800149. 25352828 PMC4196577

