# Supplementary figures and images for: A core set of venom proteins is released by entomopathogenic nematodes in the genus Steinernema
Source: PLoS Pathog. 2019 May 1;15(5):e1007626. doi: 10.1371/journal.ppat.1007626 (PMC6513111; doi:10.1371/journal.ppat.1007626)

**A)** *S. feltiae* ESP time course concentration

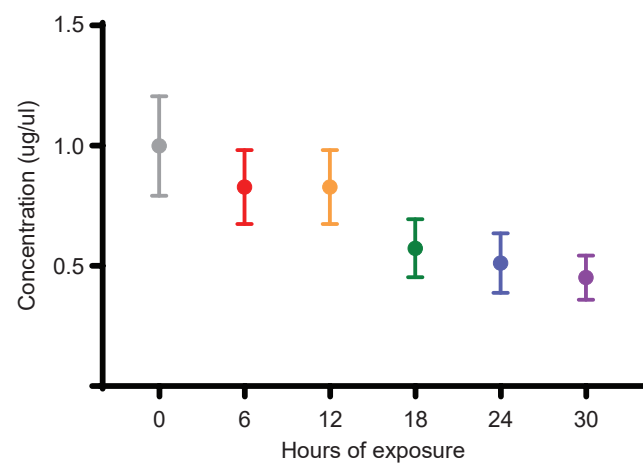

Supplement: S1 Fig — All batches were activated the same way as described in the IJ activation section of the methods and a final volume of 300 μl was collected for each time point. Each time point was repeated 3 times and the protein concentrations were determined by a Bradford assay. (PDF) [file ppat.1007626.s001.pdf]

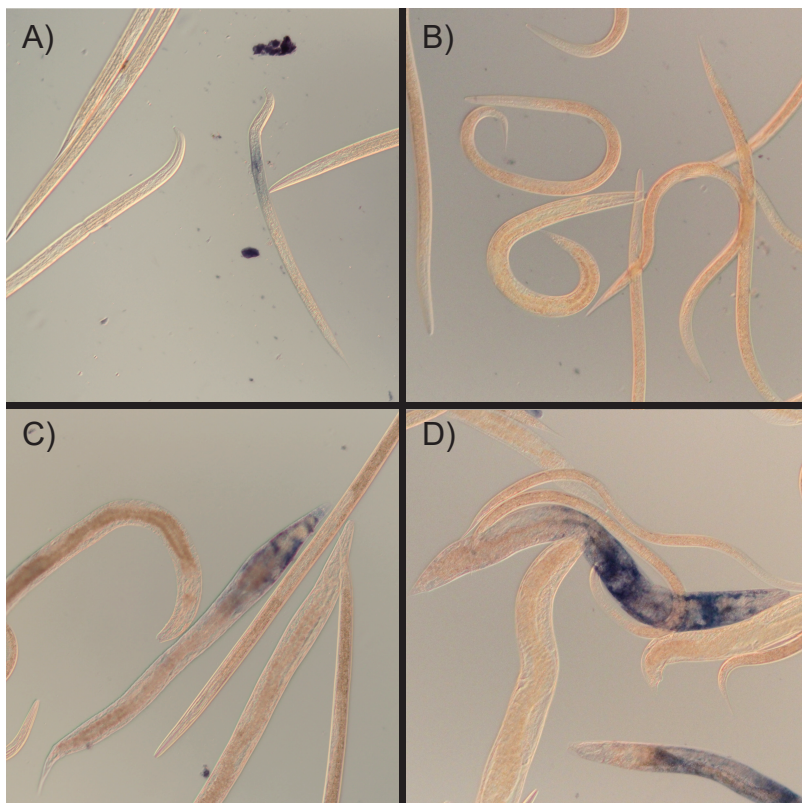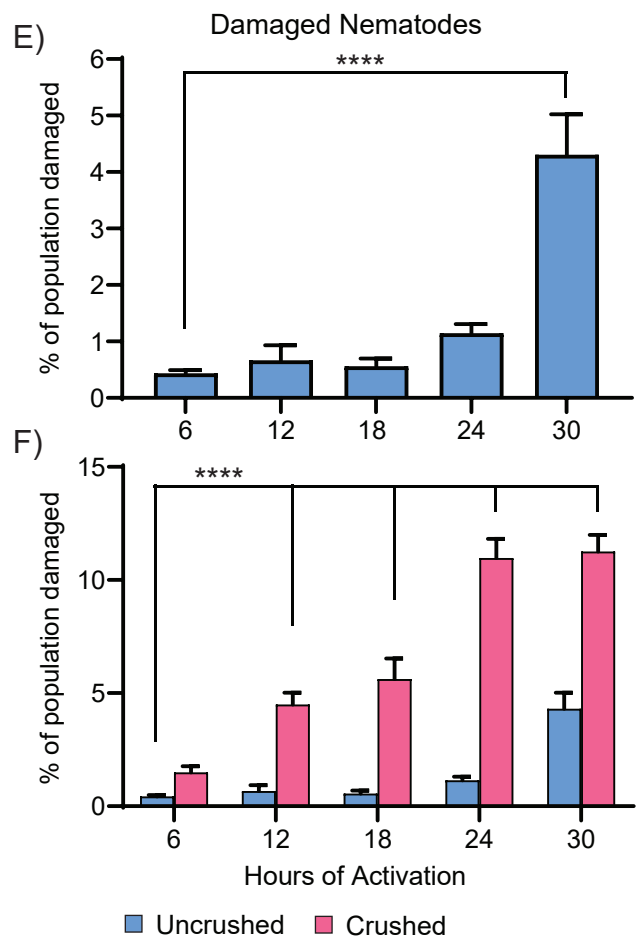

Supplement: S2 Fig — Representative pictures of damaged (indicated by trypan blue staining, 0.2% final concentration) and undamaged nematodes at various time points (A) 6 hours, (B) 18 hours, (C) 24 hours, (D) 30 hours of activation. Image B shows a view of undamaged nematodes at 18 hours while the rest show instances of damaged nematodes. (E) Percentages of the nematode population that exhibited stained damaged tissue from (uncrushed) normal sponge activation experiments. (F) Percentages of the nematode population that exhibited stained damaged tissue from manual crushing of sponge activations (pink) combined with data from panel E (blue). Bars represent the mean of 3 biological replicates with 5000 counts each and error bars represent standard deviation. **** represent statistical significance with P<0.0001. Statistical analysis was done using Graphpad Prism 8.0 software running unpaired one-way ANOVA with (recommended) Dunnett’s multiple comparisons test. The raw data counts can be found in S2 Table. (PDF) [file ppat.1007626.s002.pdf]

**A)**

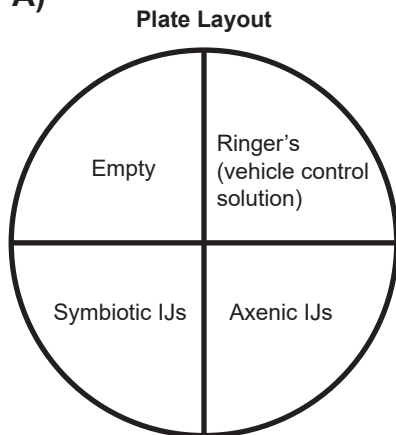

Bleach sterilized  
IJs

Hyamine sterilized  
IJs

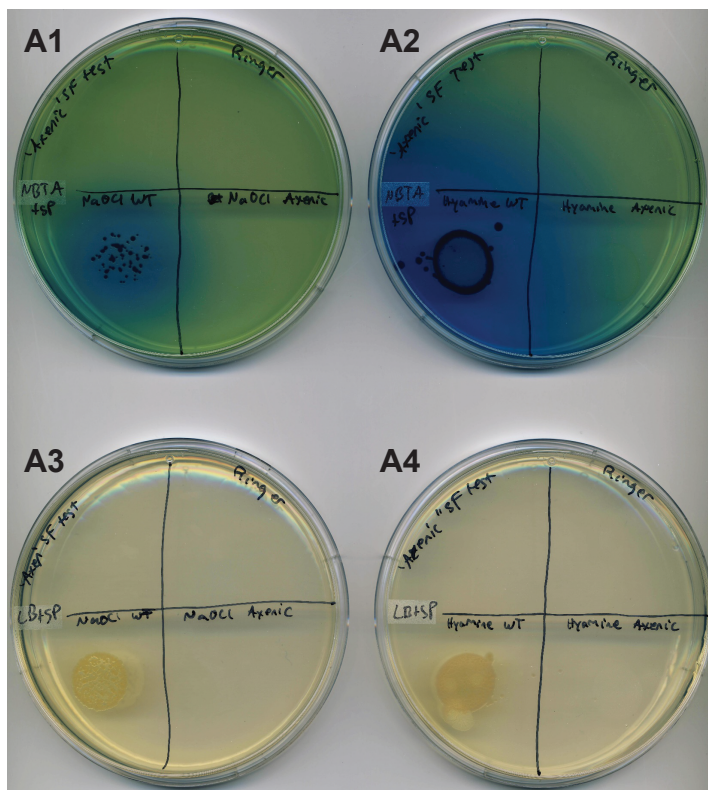

**B)**

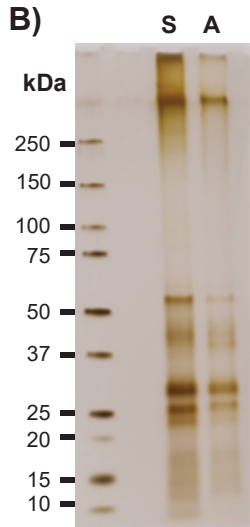

**C)**

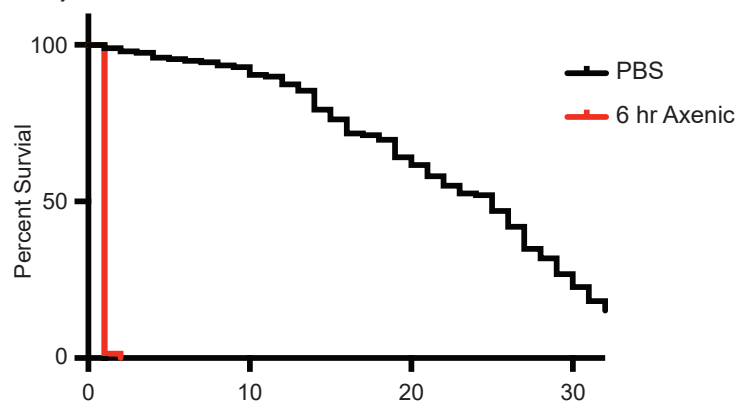

Supplement: S3 Fig — A) Schematic of how IJs were plated to assay for axenic IJs. A1) Grounded bleach surface sterilized S. feltiae IJs (symbiotic or axenic) on an NBTA plate supplemented with sodium pyruvate. Blue colonies on NBTA plates represent primary phase X. bovenii. A2) Grounded Hyamine surface sterilized S. feltiae IJs on an NBTA plate supplemented with sodium pyruvate. A3) Grounded bleach surface sterilized S. feltiae IJs (symbiotic or axenic) on an LB plate supplemented with sodium pyruvate (SP). A4) Grounded Hyamine surface sterilized S. feltiae IJs on an LB plate supplemented with sodium pyruvate. This was repeated 3 times using approximately 1000 IJs for each batch of S. feltiae IJs. B) Silver stained protein gel of ESPs collected from symbiotic (S) and axenic (A) S. feltiae IJs activated for 6 hours. C) Survival curve of D. melanogaster fruit flies injected with 20 ng of ESPs collected from axenic S. feltiae IJs activated for 6 hrs. This was repeated 3 times with at least 90 flies for reach replicate. (PDF) [file ppat.1007626.s003.pdf]

**A)**

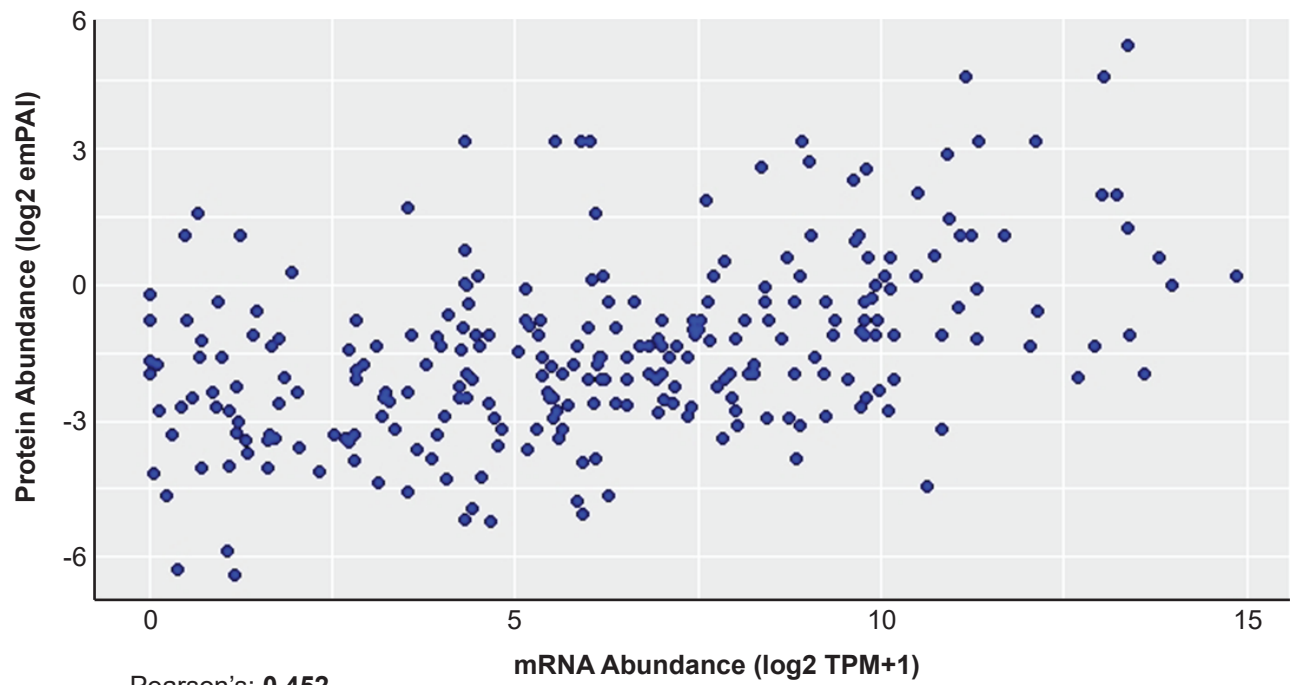

Supplement: S5 Fig — Correlation plot of mRNA abundance (log2 of TPM+1) to protein abundance (log2 of emPAI). (PDF) [file ppat.1007626.s005.pdf]

A) Number of orthologs of core ESPs in non-*Steinernema* nematodes

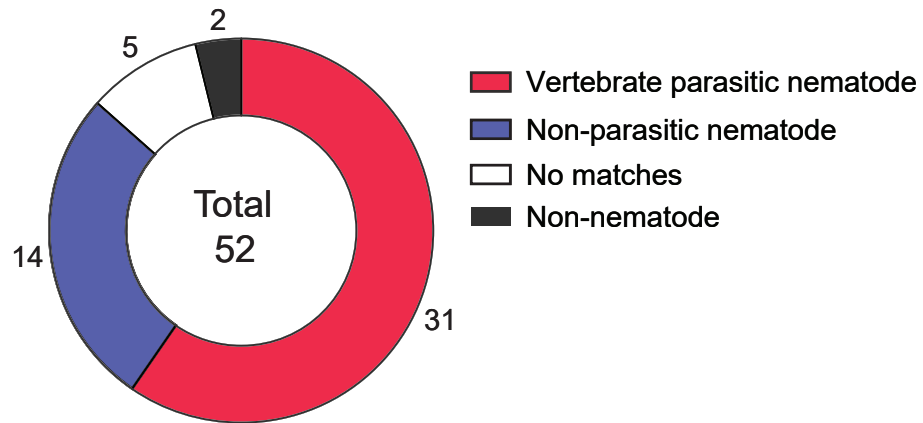

Supplement: S6 Fig — Pie chart of the 52 core ESPs which had orthologs in genera other than Steinernema and categorized into either vertebrate-parasitic nematodes, non-parasitic nematodes, or non-nematodes. The list of best orthologs found in non-Steinernema organisms can be found in S4 Table, which was produced using Blast2Go blastp default settings (E-value <1x10^-3^). (PDF) [file ppat.1007626.s006.pdf]
