# Supplementary material for: A core set of venom proteins is released by entomopathogenic nematodes in the genus Steinernema
Source: PLoS Pathog. 2019 May 1;15(5):e1007626. doi: 10.1371/journal.ppat.1007626 (PMC6513111; doi:10.1371/journal.ppat.1007626)
Supplement: S4 Fig — (A) maSigPro profiles of genes clusters during in vivo time course activation. (B) Representative GO terms for each maSigPro cluster. (C) heatmap of neuropeptide pathway enriched genes from cluster 2. (PDF) [file ppat.1007626.s004.pdf]

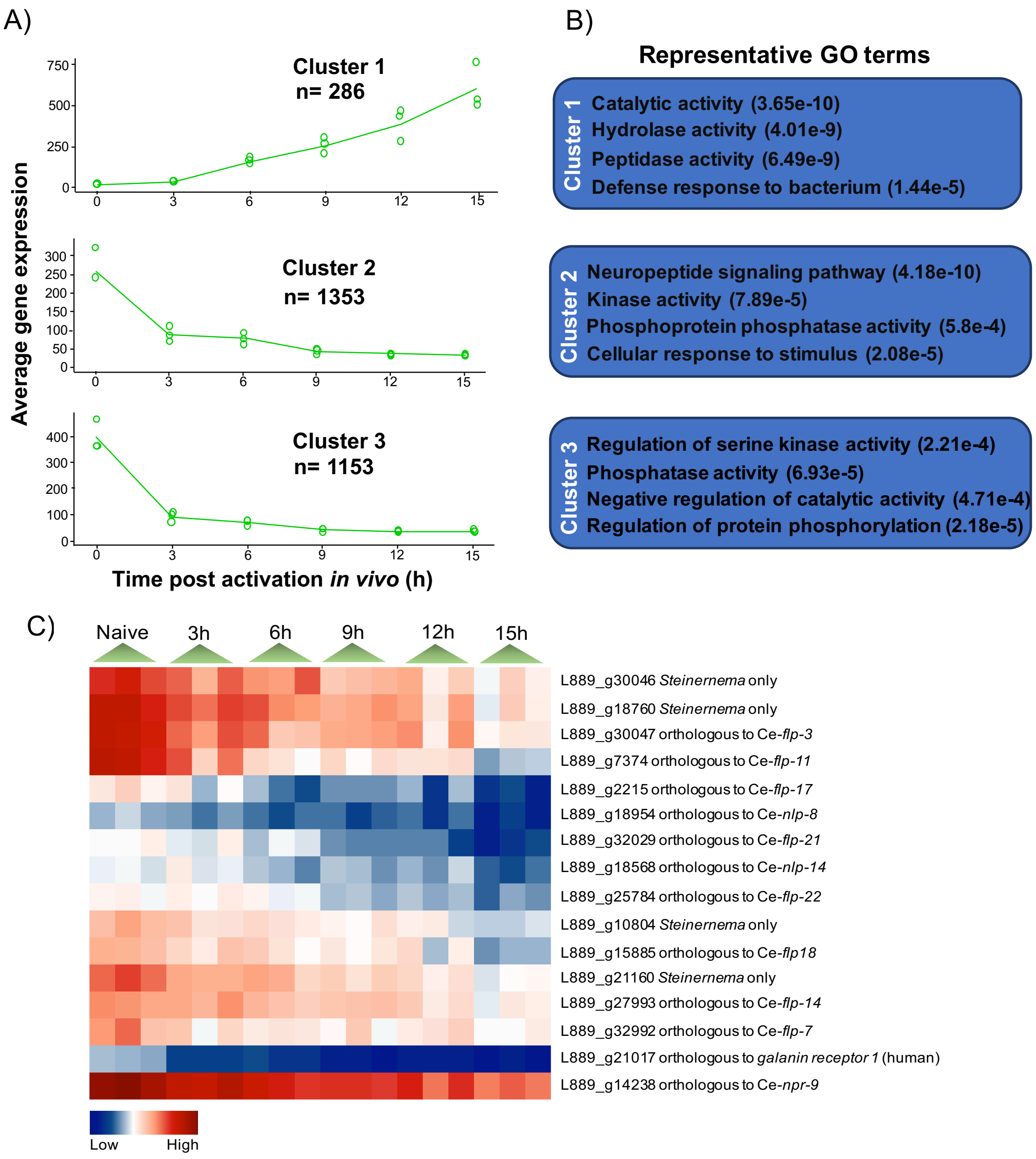
A)
Average gene expression
Cluster 1
n= 286
Cluster 2
n= 1353
Cluster 3
n= 1153
Time post activation in vivo (h)
B)
Representative GO terms
Cluster 1
Catalytic activity (3.65e-10)
Hydrolase activity (4.01e-9)
Peptidase activity (6.49e-9)
Defense response to bacterium (1.44e-5)
Cluster 2
Neuropeptide signaling pathway (4.18e-10)
Kinase activity (7.89e-5)
Phosphoprotein phosphatase activity (5.8e-4)
Cellular response to stimulus (2.08e-5)
Cluster 3
Regulation of serine kinase activity (2.21e-4)
Phosphatase activity (6.93e-5)
Negative regulation of catalytic activity (4.71e-4)
Regulation of protein phosphorylation (2.18e-5)
C)
Naive
3h
6h
9h
12h
15h
L889_g30046 Steinernema only
L889_g18760 Steinernema only
L889_g30047 orthologous to Ce-flp-3
L889_g7374 orthologous to Ce-flp-11
L889_g2215 orthologous to Ce-flp-17
L889_g18954 orthologous to Ce-nlp-8
L889_g32029 orthologous to Ce-flp-21
L889_g18568 orthologous to Ce-nlp-14
L889_g25784 orthologous to Ce-flp-22
L889_g10804 Steinernema only
L889_g15885 orthologous to Ce-flp18
L889_g21160 Steinernema only
L889_g27993 orthologous to Ce-flp-14
L889_g32992 orthologous to Ce-flp-7
L889_g21017 orthologous to galanin receptor 1 (human)
L889_g14238 orthologous to Ce-npr-9
Low
High
